# A backbone-based flow cytometry approach to decipher regulatory T cell trajectories in the human thymus

**DOI:** 10.3389/fimmu.2025.1553535

**Published:** 2025-03-03

**Authors:** Beatriz Moleirinho, Margarida Paulo-Pedro, Nicole C. Martins, Emily Jelagat, Eller Conti, Tiago R. Velho, Miguel Abecasis, Rui Anjos, Afonso R. M. Almeida, Ana E. Sousa

**Affiliations:** ^1^ GIMM- Gulbenkian Institute for Molecular Medicine, Lisbon, Portugal; ^2^ Faculdade de Medicina, Universidade de Lisboa, Lisbon, Portugal; ^3^ Cardiothoracic Surgery Research Unit, Centro Cardiovascular da Universidade de Lisboa, Faculdade de Medicina da Universidade de Lisboa, Lisbon, Portugal; ^4^ Department of Cardiothoracic Surgery, Hospital de Santa Maria, Unidade Local de Saúde de Santa Maria, Lisbon, Portugal; ^5^ Hospital de Santa Cruz, Unidade Local de Saúde de Lisboa Ocidental, Carnaxide, Portugal

**Keywords:** thymus, human immunology, regulatory T cells, T cell development, spectral flow cytometry

## Abstract

Thymus-committed regulatory T cells (Tregs) are essential for immune homeostasis. Recent findings stress their heterogeneity, suggesting possible alternate routes for thymic Treg development with unique features in humans, namely the clear evidence of Treg commitment at the double-positive (DP) stage and the presence of a significant population of CD8 single-positive (SP) FOXP3^pos^ Tregs. Here, we present a dedicated analysis strategy to a spectral flow cytometry-based study of thymus from children and aged adults (≥ 74-years-old), to further elucidate Treg development and heterogeneity in the human thymus. We applied an unsupervised analysis pipeline to data generated from 6 high-dimensional panels, taking advantage of a common backbone of 11 markers, and we were able to map thymocytes along T cell maturation stages. Generating UMAP and FlowSOM cluster coordinates from the backbone, we projected all other markers onto these, characterizing clusters with the information of all markers. Focusing this analysis on events inside a putative total Treg gate, we could portray rarer subsets of human thymic Tregs and investigate their trajectories using pseudotime analysis. We uncover clusters within human DP thymocytes uniquely expressing FOXP3 or CD25, a DP-branching trajectory towards a CD103^pos^CD8SP Tregs endpoint, and define trajectories towards CD4SP Tregs, including towards a cluster of CXCR3^pos^CD4SP Tregs, that may consist of thymic resident or recirculating Tregs, and do not expand in the elderly. Our flow cytometry approach separates Treg populations with likely distinct functions and facilitates the design of future studies to unravel the complexity of human regulatory T cells.

## 1 Introduction

Regulatory T cells (Tregs) expressing FOXP3 are essential players in T cell homeostasis, regulating autoimmune and other inflammatory responses (1, 2). Whilst Tregs may differentiate from conventional (Tconv) counterparts, the main functions in controlling autoimmunity are accomplished by thymic-generated Tregs (3, 4).

The thymic Treg heterogeneity has become a focus of recent research, driven by the development of novel animal models and advancements in single-cell transcriptomic methodologies (5–9). The uncovering of alternate pathways in Treg agonist selection, characterized by a different order in the acquisition of the Treg hallmarks CD25, the α-chain of the high affinity IL-2 receptor, and FOXP3, the lineage-associated transcription factor (5, 9), led to an update in the two-step TCR affinity-based concepts of their development (10). Current models consider that milder agonist signals drive the generation of a FOXP3-first precursor on the first step, and stronger signals generate a CD25-first precursor. The second step is mediated by γc-cytokine signals, namely IL-2 and IL-15, but a role for IL-4 and Lymphotoxin has recently been described in the regulation of FOXP3-first precursor maturation (5, 11–13). Tregs generated via each of these routes express different TCR repertoires and thus participate in the regulation of different responses (5, 12), underscoring the relevance of elucidating the signals driving their differentiation. Adding to the heterogeneity of Treg in the thymus, recirculating Tregs have also been described (14). Although FOXP3 reporter mouse models have been instrumental to shed light on many of these processes, translating these findings to human biology remains a significant challenge.

Two important considerations need to be taken into account in mouse vs. human Treg development (5, 12, 15). First, FOXP3 expression is found at much higher frequencies in human double-positive (DP) thymocytes than in mice (6, 15–17), hence human Tregs may mainly commit at DP (18, 19) rather than at the single positive (SP) stage. Second, CD8^pos^FOXP3^pos^ Tregs, whose relevance is gaining attention (20), are found at much higher frequencies in the human thymus compared to mice (15, 18, 21). The intricacies in human thymic Treg development and heterogeneity are far from being elucidated (22).

The advent of spectral flow cytometry enabled the use of more complex panels with overlapping spectra, resulting in an increase in the number of markers analyzed simultaneously (23, 24). When combined with emerging technologies such as CITE-Seq, single-cell transcriptomics, and spatial transcriptomics, these advances drive to a deeper understanding of thymic biology (6–8, 25, 26). However, many of these methodologies are resource-intensive, and spectral flow cytometry is likely to remain the primary approach for a substantial proportion of studies due to its accessibility and versatility. To handle the high-dimensional data generated, unsupervised clustering algorithms are being increasingly used to identify potential subpopulations based on the simultaneous expression of multiple markers. Furthermore, trajectory inference methods are providing valuable insights into cellular differentiation pathways (27, 28).

Here, we applied a flow cytometry-based approach to investigate human thymic Treg heterogeneity, using a common backbone strategy and adapting existing R-based algorithms. We integrated data from 6 different spectral flow cytometry panels to analyze thymic Tregs expressing FOXP3 and/or CD25, allowing us to define subpopulations and investigate lineage trajectories. In addition, we compared the relative distributions of the identified subsets in the thymus from children in parallel with older individuals. Altogether, we describe a strategy for integrating flow cytometry data from multiple panels combined with trajectory inference, enabling us to help decipher Treg development in the human thymus.

## 2 Materials and methods

### 2.1 Human samples

Human thymic specimens, from newborns to 15-year-old children, were obtained from routine thymectomy performed during pediatric corrective cardiac surgery at Hospital de Santa Cruz - Unidade Local de Saúde de Lisboa Ocidental, following parental written informed consent. Thymic specimens from adults older than 74-years-old were obtained from patients undergoing cardiac surgery for coronary artery disease or severe aortic stenosis at Hospital de Santa Maria – Unidade Local de Saúde de Santa Maria, after patient written informed consent. Samples used in the manuscript are listed in **Supplementary Table S1**. The thymic samples would be otherwise discarded. The study was approved by the Ethical Boards of the Centro Académico de Medicina de Lisboa, and of the Hospital de Santa Cruz, Portugal.

### 2.2 Sample processing

Thymic samples were maintained at 4°C and processed within less than 24h upon collection. Total thymocytes were recovered through tissue dispersion using a 70μM filter (BD Biosciences) and a syringe plunger and subsequently isolated on a Ficoll-Paque™ Plus (Cytiva) density gradient, as previously described (18). Thymic tissue with blood was discarded to avoid cross-contamination with peripheral lymphocytes.

### 2.3 Flow cytometry

**Figure 1:**
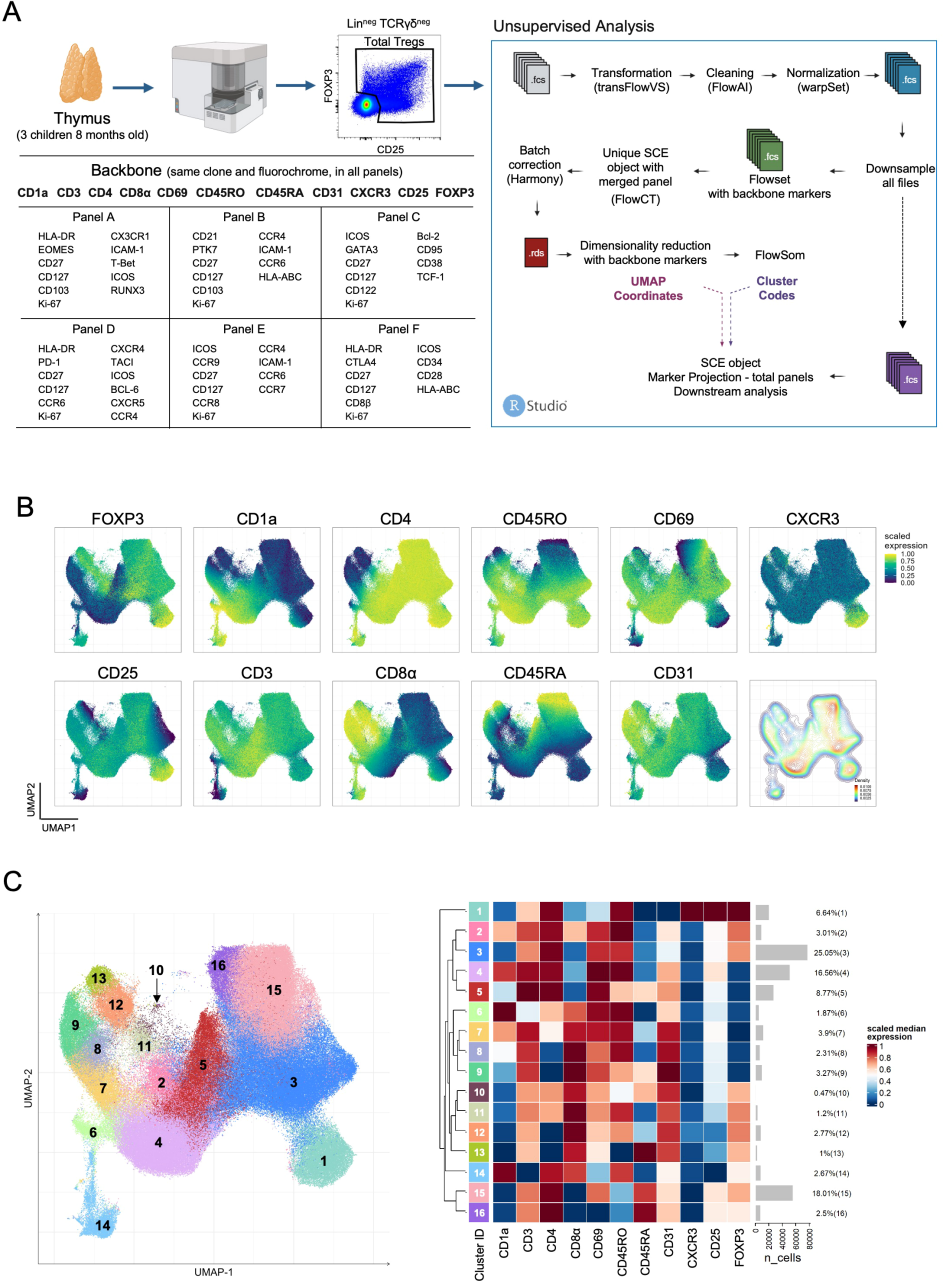
Backbone strategy for panel integration in flow cytometry analysis of human thymic Tregs. **(A)** The scheme depicts on the left the experimental design, flow cytometry panel markers, and gating strategy to select total putative Tregs, and on the right the unsupervised analysis pipeline for file processing and panel integration. **(B)** UMAP of human thymic Tregs showing scaled expression of each backbone marker and a density plot (bottom right). **(C)** UMAP of human thymic Tregs (*n=*311.224), colored by clusters (left) with the heatmap of scaled median expression of backbone markers from each cluster with gray bars showing the numbers of cells on each cluster along the respective proportion within total Treg thymocytes (right) (see also **Supplementary Figures S1-S5**). Created in BioRender. Biorender, I. (2025) https://BioRender.com/j67h095.

Thymocytes isolated from 8-month-old infants (n=3) were simultaneously stained for 6 different panels (panels A to F), sharing a common backbone (**Figure 1A**; **Supplementary Table S2**). A backbone was defined from markers sharing the same fluorochrome for the same antigen (clone). Thymocytes from older patients (≥ 74-year-old, n=3) were processed only for panel F, as the obtained cell numbers were insufficient to do more than one panel. Surface staining was performed on fresh thymocytes for 25 minutes at room temperature, and were fixed, permeabilized, and stained using the eBioscience™ Foxp3/Transcription Factor Staining Buffer Set (eBioscience), as per manufacturer’s instructions, for intracellular staining. Samples were acquired on a 3-laser Cytek™ Aurora (Cytek Biosciences, Fremont, California) spectral flow cytometer and analyzed as outlined in **Figure 1A**.

### 2.4 Pre-processing

All FCS files were unmixed and manually compensated in SpectroFlo software (v3.2.1; Cytek Biosciences, Fremont, California) or FlowJo (v10.8.1; BD Biosciences, Franklin Lakes, New Jersey), followed by the gating strategy outlined in **Supplementary Figure S1** using FlowJo (v10.8.1). Thymocytes were gated based on forward and side scatter, doublets were excluded by plotting the area and height of forward and side scatter, live cells were gated by live/dead staining, and non-thymocytes were excluded by lineage markers (CD11c, CD14, CD16, CD19 and CD123). TCRγδ T cells were also excluded, included in the lineage channel in panels A-E, or on a separate channel in panel F. For Treg cells, a further gate based on FOXP3 and CD25 expression was applied. Gated samples were exported as FCS files and imported into R (v4.3.3; R Foundation for Statistical Computing, Vienna, Austria) as flowSets (*flowCore* v2.14.2). Arcsinh transformation was applied to all samples (*FlowVS* v1.34.0). After transformation, automatic quality control based on flow rate, signal acquisition and dynamic range was performed (*flowAI* v1.32.0). To correct for technical intersample variance, normalization was applied using fdaNorm (*FlowStats* v4.14.1). Upon pre-processing, samples were exported as FCS files (*flowCore* v2.14.2).

### 2.5 Panel-integration strategy

Pre-processed FCS files (samples from panels A to F) were uploaded to R and downsampled to the minimum number of events across all samples (322.259 for total thymocytes, 17.442 for Treg and 248.748 for Tregs in the comparison of young and older human thymi). Downsampling was performed randomly and the resulting flowSets were exported as FCS files. Files were reimported as flowSets, one for each panel, and subsetted to backbone markers only. FlowSets were merged, and FCS files for each sample only with backbone markers were exported. Batch correction was performed for technical panel variance. We compared normalization by warp and gauss with batch removal through Harmony, as implemented by the *FlowCT* (v1.0.0) package. Harmony was selected as the best overall method.

### 2.6 Dimensionality reduction and cell clustering

For UMAP (*uwot* v0.1.16), backbone marker expression levels (batch corrected with Harmony) were used (**Supplementary Figures S2, S3**). The number of neighbors and minimum distance were tested and defined. Visualization was achieved by plotting the marker expression values scaled from 0 to 1 on the UMAP (*CATALYST* v1.26.1 and *ggplot2* v3.5.1). Cells were clustered using FlowSOM (*CATALYST* v1.26.1) and visualized in a cluster tree (*clustree* v0.5.1). The number of clusters was decided based on tree stabilization, and phenotypically similar clusters were manually merged to avoid over-clustering (**Supplementary Figure S4**). The single-cell experiment was exported as an R Data Format (RDS). Clusters of cells lacking marker expression, which separated early in the tree, were excluded. The diffusion map was implemented as in the *destiny* package (v3.16.0).

### 2.7 Pseudotime

Trajectory inference was performed with *Slingshot* (v2.10.0) on a diffusion map representation of the flow cytometry data, which captures continuous transitions between cell states. We used the clusters previously established using FlowSOM and defined cluster 14 as the starting cluster based on its high expression of CD1a, CD4 and CD8 and lower CD3 expression, and then *Slingshot* was used to fit smooth trajectories through the high-dimensional space. Final clusters (9, 13, 16, 1 and 5) were defined based on their high expression of CD45RA and/or their location at the extremities of the diffusion map. Finally, each cell is assigned a pseudotime value, indicating its relative position along the inferred trajectory. Cells at earlier stages have lower pseudotime values, while more differentiated cells have higher values. Visualization was achieved by plotting the inferred pseudotime values and scaled marker expression on the diffusion map (*ggplot2*). The single-cell experiment was exported as an RDS.

### 2.8 Marker visualization

The visualization of the markers not included in the backbone was performed by importing the FCS files after event down-sampling from a particular panel containing all markers’ information and the RDS file (before cluster removal and diffusion map generation), followed by subsetting to the particular panel. UMAP coordinates and cluster codes were extracted and provided to the complete dataset. Events from previously removed clusters were again excluded. Diffusion map coordinates, lineages, and curves were extracted from the RDS and applied to the dataset. Marker visualization was conducted as previously described, both on the UMAP and the diffusion map.

### 2.9 Cell culture

Fresh thymocytes, isolated from 7-month-old to 1-year-old infants (n=3), were used to assess cytokine production after stimulation with phorbol myristate acetate (PMA; 50ng/mL, Sigma-Aldrich) plus ionomycin (500ng/mL; Sigma-Aldrich), in the presence of brefeldin A (10µg/mL; Sigma-Aldrich) for 4 hours, followed by surface staining, fixation, permeabilization, and intracellular staining, as previously described (29).

### 2.10 Software and algorithms used

All software and algorithms used can be found in **Supplementary Table S3**, together with the relevant references.

## 3 Results

### 3.1 Integrating data from multiple spectral flow cytometry panels to investigate Treg heterogeneity in the human thymus

Spectral flow cytometry allows the expansion of the number of markers in a single-tube. When combined with the potential of the new analysis algorithms it offers powerful tools for detailed, single-cell analysis, yet to be fully explored. We took profit of existing algorithms in the R platform and designed an approach to help decipher thymic Treg heterogeneity in the human thymus. In order to identify and characterize relevant stages from intermediate developmental stages to mature Treg subpopulations, we used a 3-laser spectral flow cytometer and an unsupervised analysis approach. We developed a panel-integration strategy, based on a common backbone approach and an R-studio pipeline (**Figure 1A** and methods section), adapting existing algorithms. Three thymuses from 8-month-old children undergoing cardiac reconstructive surgery were analyzed (**Figure 1A**). We selected events inside a total thymocyte gate defined by the expression of FOXP3 and/or CD25 to include all putative Treg-lineage cells (**Figure 1A**; **Supplementary Figure S1**). The common backbone of 11 markers, using the same antibody clones and fluorochromes, was designed to contain, besides the Treg-lineage, markers able to discriminate major T cell developmental stages (CD1a, CD3, CD45RO, CD45RA, CD69, CD31, CD4, and CD8), and CXCR3, a differentiation marker potentially associated with recirculating cells (**Figure 1A**; **Supplementary Figure S2**). Each panel (A-F) included other non-backbone markers potentially informative on thymic Treg subsets, allowing us to gain information on a total of 44 markers (**Figure 1A**). The analysis pipeline is also outlined in **Figure 1A**. The initial “raw”-FCS files were transformed, cleaned, and normalized using available R-packages (detailed in Methods section) before downsampling, upon which two different FCS files were created: one (green in **Figure 1A**) including only data from the backbone markers, used to generate UMAP coordinates and FlowSOM clustering, and a second one (purple in **Figure 1A**), including all marker information from each tube that can then be projected in the UMAP coordinates and the identified clusters.

The UMAP generated with information from backbone markers allowed us to evaluate the putative total Tregs across the main developmental stages in the human thymus (**Figure 1B**). UMAP regions became apparent, such as cells expressing just one of the Treg-lineage markers (FOXP3^pos^CD25^neg-low^, CD25^pos^FOXP3^neg-low^), and DP, CD4SP and CD8SP thymocytes. Also observed was a peninsula of CXCR3 expressing CD4SP Tregs (**Figure 1B**). The density plot allowed us to visualize the relative paucity of immature cells, with lower CD3 expression and high expression of CD1a, CD4 and CD8α, as well as of CD8SP Tregs (**Figure 1B**). These were not observable in the UMAP performed in parallel using total thymocytes (**Supplementary Figure S2**), highlighting the advantage of the focused putative Treg analysis. Importantly, the expression of backbone markers from each panel was virtually equal in all panels (**Supplementary Figure S3**), indicating the reproducibility of signal from the different panels and illustrating the successful panel-integration.

Next, we used the information of the backbone-marker-only FCS files to apply FlowSOM clustering (see Methods). The clustree visualization led us to eliminate two small clusters, completely separated in the tree from the onset, and merge other minor clusters (**Supplementary Figure S4**). All 16 clusters were present in each of the three individual samples with only slight frequency variations (**Supplementary Figure S5**). The 16 identified clusters were then projected onto the UMAP with the scaled expression of the backbone markers in each cluster depicted in the heatmap in **Figure 1C**. In addition to the classical Treg clusters, expressing high levels of CD25 and of FOXP3 (clusters 12, 13, 15, 16) several emerged that deserved exploration. Clusters 4 to 9 lacked FOXP3 expression or expressed it at very low levels, and thus was difficult to exclude the presence of Tconv. These included CD4SP (clusters 4-5), CD8SP (clusters 8-9), and DP thymocytes (clusters 6-7), the latter displaying the highest CD1a expression, in accordance with an immature state. Conversely, clusters 11-14 express very low to no CD25 and are either CD8SP (clusters 12-13) or DP (clusters 11 and 14). From the latter, cluster 14 stands out as expressing the lowest levels of CD3, highest of CD1a and low CD69, denoting the possibility of representing the most immature cells in the gate. In contrast, cluster 11 appears to be gaining expression of CD25. The CD4SP cluster 1 appeared separated in the UMAP, displaying marked CXCR3 expression along with CD45RO, low levels of CD45RA or CD31, but interestingly, some CD1a and CD8α, suggesting the inclusion of immature cells (**Figures 1B, C**). Overall, the unsupervised analysis of the backbone markers’ expression unveils important heterogeneity within putative total Tregs in the human thymus, raising questions concerning relationships between subpopulations and developmental trajectories.

**Figure 2:**
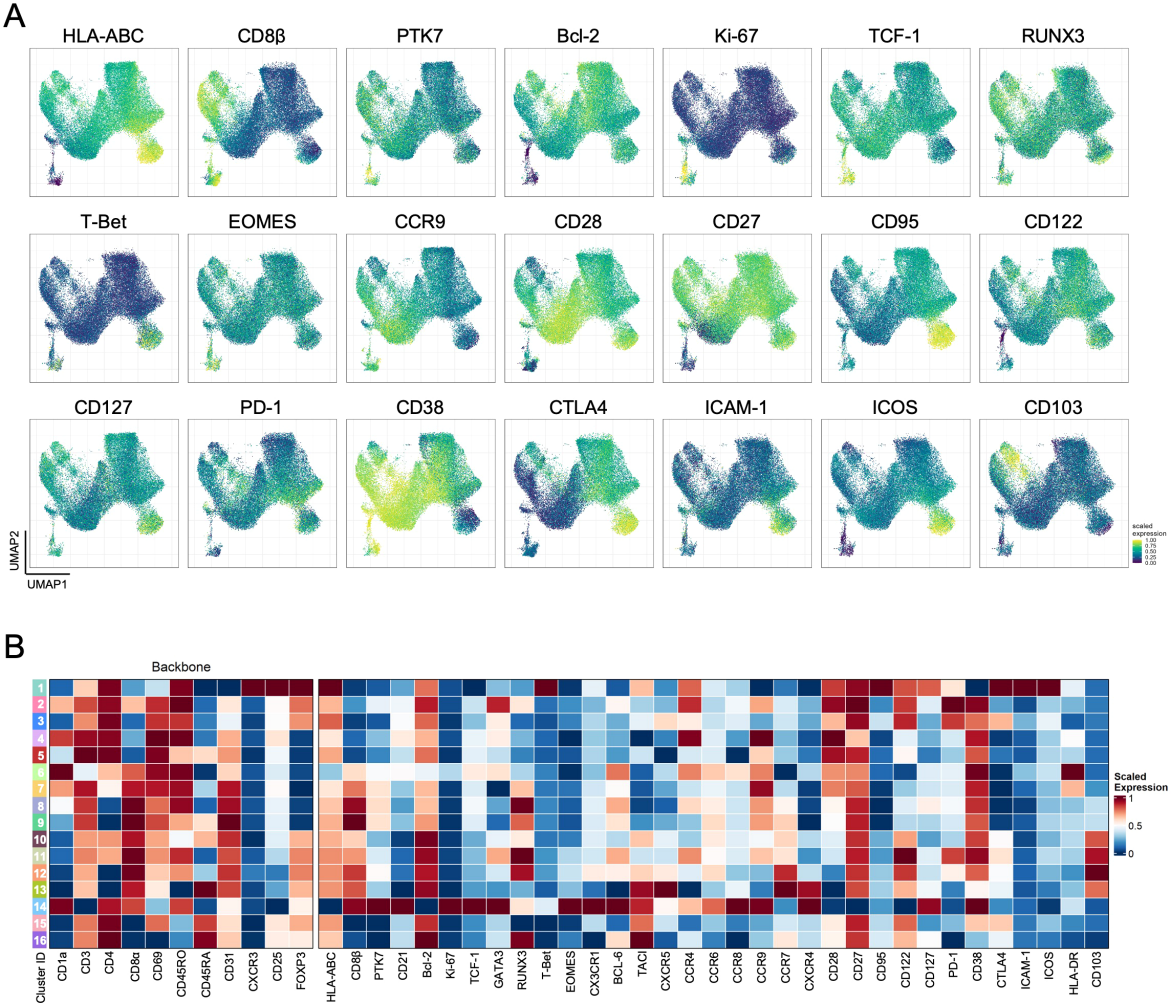
Characterization of total Tregs in the human thymus. **(A)** Scaled expression of additional markers not included in the backbone markers projected on the UMAP coordinates generated with backbone markers. **(B)**. Heatmap of scaled median expression of the backbone and all other additional markers in each of the 16 clusters defined using FlowSOM (see also **Supplementary Figure S6**).

We have then searched to use the information from all other markers to shed light on these aspects, taking advantage of the panel-integration strategy. Our methodology allowed us to display the expression of all other markers from the six panels projected onto the UMAP (**Figure 2A**; **Supplementary Figure S6**) and then associate their expression with the FlowSOM-derived clusters (**Figure 2B**). The DP cluster 14 expressed the highest levels of the proliferative marker Ki-67, the Wnt signaling-associated transcription factor TCF1, and the cortical-associated chemokine receptor CCR9, together with the lowest expressions of HLA-ABC, CD27, CD28 or CCR7, attesting the more immature nature of these cells (**Figure 2**; **Supplementary Figure S6**). Interestingly, this phenotype is partially shared by the DP cluster 6, which did not express FOXP3 (**Figure 2**; **Supplementary Figure S6**). However, this CD25-only cluster featured much lower levels of Ki-67, and some CD69 expression alongside the brightest HLA-DR (**Figure 2**; **Supplementary Figure S6**), possibly betraying a stronger TCR-signal strength perceived.

The other low-null FOXP3 clusters, CD4SP (clusters 4-5) and CD8SP (clusters 8-9) thymocytes, differed mostly in the symmetrical expression of CD45RO and CD45RA, suggesting that these represent consecutive thymocyte developmental states in each of the SP populations, with RUNX3 being more expressed in the latter (**Figures 1**, **2**), as expected due to its association with the CD8 lineage (30). Importantly, they all expressed low-null CTLA-4, an important suppressor marker (31), and intermediate levels of the α-chain of the IL-7 receptor, CD127 (**Figure 2**), further suggesting that these clusters may include Tconv lineage cells. On the other hand, the clusters expressing FOXP3 and low-null levels of CD25 (clusters 10-13) were all CD103-expressing (**Figure 2**). These included DP and CD8SP thymocytes, supporting a role for CD103 in CD8 Treg development from the DP stage (15, 32).

FOXP3^pos^CD25^pos^ CD4SP (clusters 15-16) and CD8SP (clusters 12-13) populations represented a significant fraction of events, as expected (**Figure 1C**; **Supplementary Figure S5**), differing in the gradual changes in expression levels of different markers and on the higher levels of PD-1 and RUNX3 within the CD8SP clusters (**Figure 2**; **Supplementary Figure S6**). These subtle differences contrast with the dramatic immunophenotype found in the CXCR3-expressing cluster 1, displaying the highest levels of the Treg lineage markers CD25, FOXP3, and CTLA-4 in parallel with CD127, activation-associated markers such as CD95 and ICOS, as well as the adhesion molecule ICAM-1 (**Figure 2**), in the absence of significant expression of other chemokine receptors associated with T cell differentiation (**Supplementary Figure S6**). Of note, this cluster also expressed T-bet (**Figure 2**), linked to CXCR3 expression, but there was no evidence of IFN-γ production in a complementary stimulation assay (**Supplementary Figure S7**). Intriguingly, some of these cells also expressed, albeit at low levels, CD8 (α and β) and CD1a, suggesting the inclusion of some developing thymocytes in this cluster and thus that not all these cells are CD4SP (**Figure 1**, **2**). Of remark, although the manual analysis recapitulates all these findings, as illustrated in **Supplementary Figure S8**, our panel-integration approach allows a deep Treg profiling in the human thymus, raising relevant questions about their developmental relationships.

### 3.2 Trajectory inference using a backbone flow cytometry approach to investigate Treg development in the human thymus

**Figure 3:**
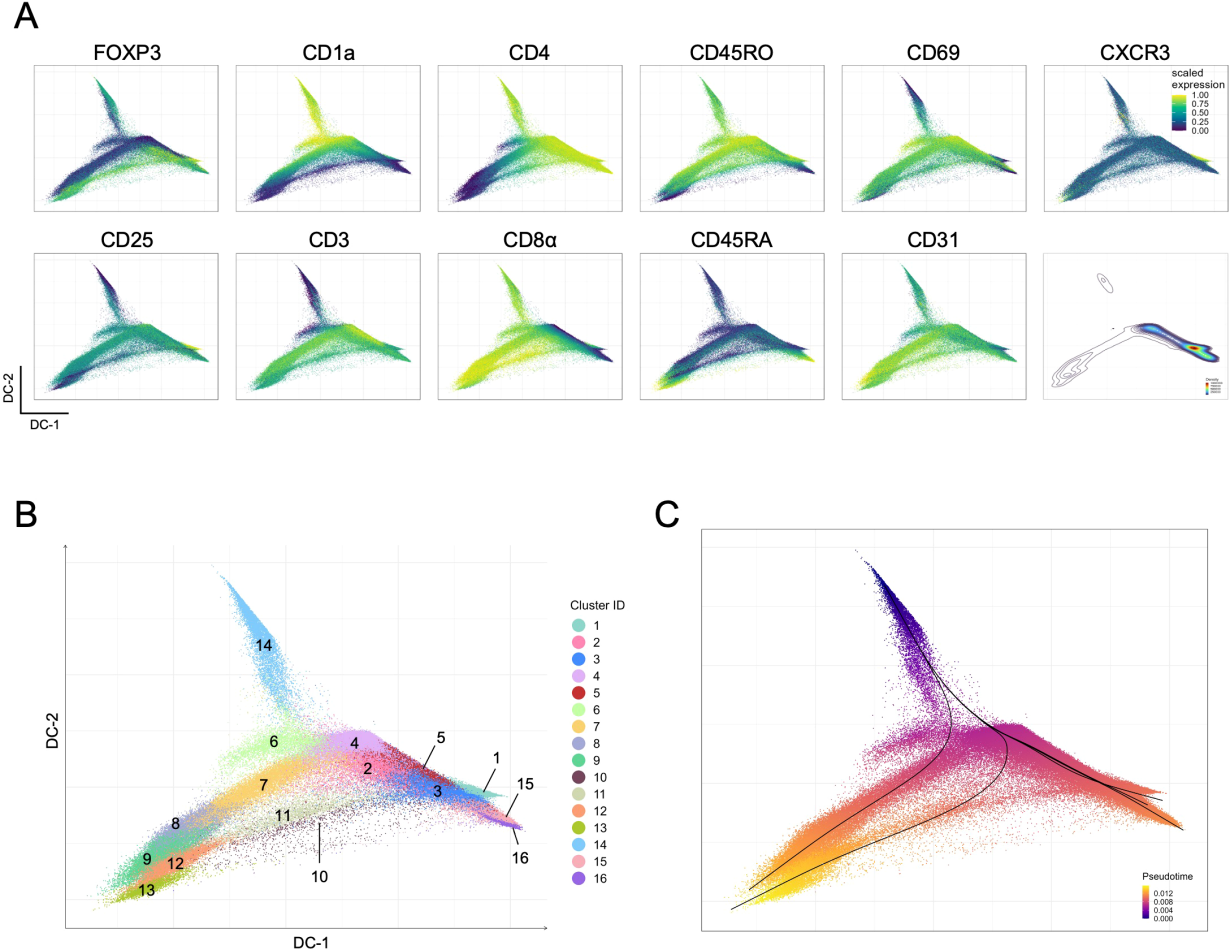
Trajectory inference for total Tregs in the human thymus. **(A)** Projections of the scaled expression of the backbone markers in the diffusion map generated with backbone data from the merged panels; the distribution of events is also shown (bottom right). **(B)** Diffusion map projection generated with backbone marker expression, colored by FlowSOM clusters. **(C)** Pseudotime trajectory inference, showing the five identified trajectories (lines) (see also **Supplementary Figure S9**).

**Figure 4:**
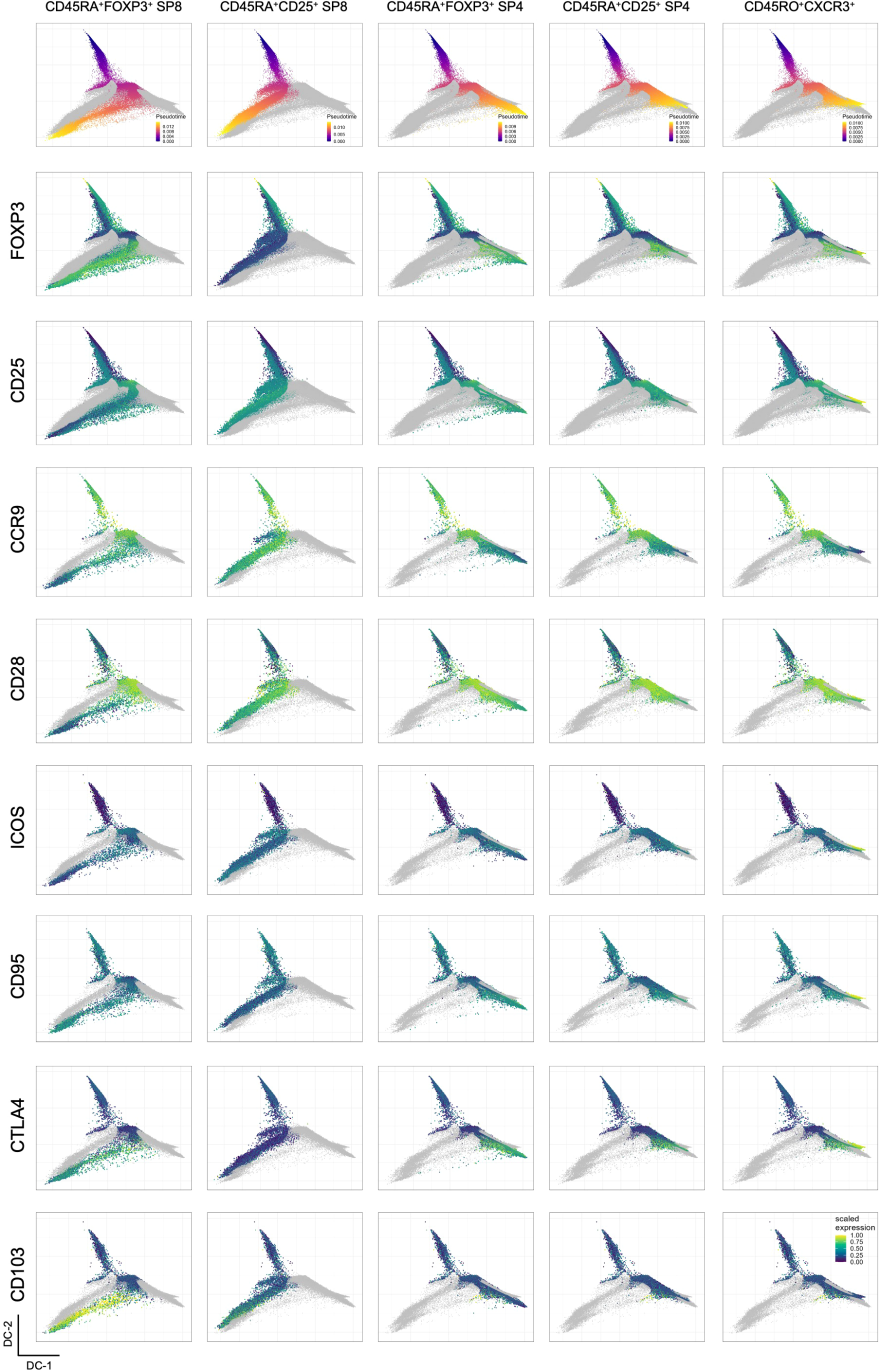
Analysis of inferred Treg trajectories in the human thymus. Projection of each inferred Treg trajectory labeled at the column top in the diffusion map generated with backbone data, showing events in each lineage colored by pseudotime values (first row) and the projection of the scaled expression of selected markers, including backbone and non-backbone markers in the subsequent rows.

Our results further support alternate paths of Treg development in the human thymus (5–7, 12), leading us to take advantage of our approach to explore available trajectory algorithms. We employed trajectory inference with pseudotime projection (see Methods) onto diffusion maps defined according to coordinates generated with the backbone marker information (**Figure 3A**). This allows the projection of all markers from panels A to F (**Supplementary Figure S9**), as well as the 16 identified clusters (**Figure 3B**). The high expression of CD1a and low expression of CD3 (**Figure 3A**), corresponding to cluster 14 (**Figure 3B**), presents as a tip of the diffusion map and was used as the point of origin for the pseudotime trajectory inference analysis (**Figure 3C**). The CD45RA-expressing clusters and/or clusters located at extremities (clusters 9, 13, 16 and 5) of the diffusion map were considered endpoints, plus an additional one corresponding to the CXCR3-expressing cluster 1, also at an extremity (**Figure 3**). Overall, we visualized five trajectories (**Figure 3C**) that could be further characterized by projecting markers from panels A to F (**Figure 4**).

Three trajectories corresponded to CD4SP endpoints, on the right side of the diffusion map and collecting a large part of events (**Figure 3A**, density plot, and **Figure 3C**), and to two CD8SP endpoints, on the left side of the diffusion map (**Figures 3A, C**).

Focusing on the CD8SP clusters, it becomes apparent that the determinant markers in these two divergent trajectories were the expression of FOXP3 and CD103 *versus* CD25 expression (**Figure 4**). In addition, CD8SP CD25-only lacked CTLA-4 and featured a less mature phenotype, with sustained expressions of CD1a and CD28, and higher levels of CD38 at the endpoint (**Figure 4** and **Supplementary Figure S9**). This visualization and analysis further interrogate the Treg-lineage commitment of CD25-only CD8SP.

Concerning CD4SP endpoints, three different trajectories were identified, including a CD25-only (**Figures 3C**, **4**). However, contrasting to CD8SP, a clear marker distinction was not found, which suggests a possible overlap of truly CD25-only with CD25^pos^ CD4SP that later acquired FOXP3 expression. Finally, the CXCR3^pos^ CD4SP trajectory showed more concordance with the one expressing FOXP3 than the CD25-only trajectory (**Figure 4**).

Thus, combining trajectory inference with our panel-integration approach further distinguishes different Treg populations that likely follow distinct developmental pathways in the human thymus.

### 3.3 Probing the Treg heterogeneity in the aging human thymus

**Figure 5:**
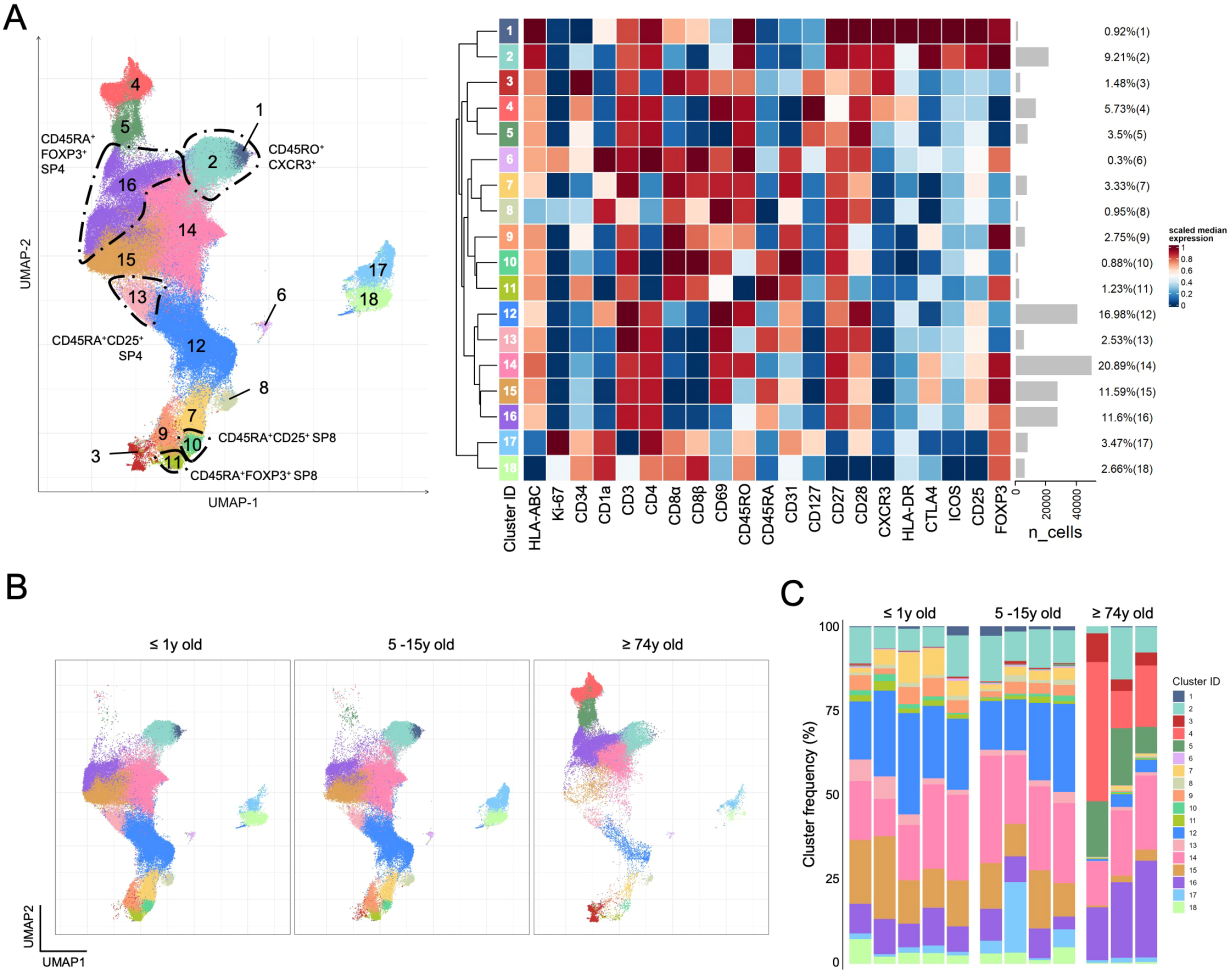
Comparison of total Tregs from young and older human thymus. **(A)** UMAP was generated using total Tregs from the thymus of ≤ 1 year old children (n=5), older children of 5-15 years of age (n=4), and older adults ≥ 74 years old (n=3) analyzed with panel F (**Figure 1**), followed by FlowSOM clustering (*n=*241.574); the 18 considered clusters are projected onto the UMAP (left) with the heatmap displaying the scaled median expression and the gray bars depicting the number of events on each cluster with their respective proportion (right); manual annotation highlights the clusters corresponding to the endpoints identified in trajectory inference analysis. **(B)** Cluster projection on the UMAP of Tregs according to age groups of thymus donors. **(C)** Stacked bar graphs depict the distribution of cluster frequencies in each thymus with the age group identified at the top. (see also **Supplementary Figures S11, S12**).

Next, we investigated whether the subset distribution was altered with age, providing further clues for understanding human thymic Treg biology. For this, we analyzed thymic tissue from three representative age groups, namely collected from individuals aged 74 years or older (n=3) undergoing cardiac surgery, older children and male adolescents (5-15 years old, n=4), and children below 1-year-old (n=5) (**Figure 5**). As expected, the total number of cells recovered in the older thymi was low, imposing the selection of only one of the panels in **Figure 1** (Panel F) to ensure the acquisition of a high number of putative Tregs (**Supplementary Figure S10**). We applied the same unsupervised analysis pipeline, minus the panel-integration process as only panel F was used. The UMAP generated with total thymocytes (**Supplementary Figure S11A**), and with total Tregs (**Supplementary Figure S11B**) allowed the visualization of markers and the identification of areas corresponding to the subsets and trajectories from the analysis using the 6 panels. FlowSOM clustering led us to identify 18 clusters (**Supplementary Figure S12**) that were projected onto the UMAP (**Figure 5A**). The clusters that likely correspond to the 5 trajectory endpoints from **Figures 3** and **4** are manually annotated in **Figure 5A**. The analysis of the cluster distribution projected onto the UMAPs of the concatenated putative Tregs from individuals according to the three age classes showed relative stability during childhood and adolescence with marked alterations with aging (**Figure 5B**). This was even clearer in the individual analysis of cluster distribution provided in **Figure 5C**. Aging was associated with an apparent expansion of CD25-only cells with a unique phenotype (clusters 3, 4, and 5), rare in the younger samples. These cells were CD25^low^, lacking FOXP3 and CTLA-4, expressing CD45RO in the full absence of CD45RA, and high levels of CD127, CD69 (clusters 3-4) and HLA-DR (cluster 4), a phenotype compatible with locally activated or recirculating cells. Additionally, the most immature CD1a expressing clusters (clusters 1; 6-8; 12; 17-18) appeared reduced, even when considering the relative expansion of clusters 3-5. Also striking was the almost absence of CD8SP Tregs expressing FOXP3 with low levels of CD25 in the older thymi (clusters 9 and 11). The lack of CD103 in panel F precluded a detailed analysis of these CD8SP Tregs. Finally, the CXCR3^pos^ CD4SP Tregs were divided in this analysis into two clusters (clusters 1 and 2), mostly differing in the expression of CD8α and CD8β, as well as of CD1a (**Figure 5A**). Interestingly, cluster 1, expressing these markers, was virtually absent in the older thymus samples, and the cluster 2 was not expanded. These findings favor a recent thymic origin for at least some of these CXCR3^pos^ CD4SP cells and argue against the recirculating hypothesis.

In conclusion, all the identified subsets were conserved in the human thymus from infancy to old age. The study of older subjects represents an important strategy to better understand Treg biology, their heterogeneity and developmental paths in the human thymus.

## 4 Discussion

The heterogeneity of the thymic Tregs and its biological implications have been increasingly recognized (4, 5, 7–9, 12, 28, 33), raising important questions concerning mechanisms involved and the need to uncover markers for their distinction. We designed an experimental approach to profile Tregs in the human thymus leveraging the potential of spectral flow cytometry and the advances in algorithms for unsupervised analysis. Focusing our analysis of putative total Tregs, including all FOXP3 and/or CD25 expressing thymocytes, we uncover Treg subsets associated with distinct developmental paths. Moreover, we show that these subsets were conserved during aging.

The spectral flow cytometry could be applied flexibly and using fewer resources than CyTOF and sequencing-based methods (23, 24, 34). This is particularly relevant for subpopulation sorting for mechanistic and fate-mapping studies, ultimately required for functional validation. Our backbone marker approach allowed leveraging information from 44 potentially co-expressed markers distributed into six distinct panels to help decipher Treg heterogeneity in the human thymus using a 3-laser flow cytometer. The common backbone markers, allowing the classification of thymocytes along known developmental stages and the total Treg identification, were used to generate a UMAP where the expression of the other markers was projected. Importantly, we have not attempted an “imputation” of values from markers not included in the same panel or tube (35) to characterize populations or subsets according to non-backbone marker expression. Instead, we generated backbone-based UMAP and FlowSOM cluster coordinates to project and infer cluster characterization using all markers across 6 panels, a strategy similar to one previously used with CyTOF data (34). While this methodology does not allow the attribution of an expression value for a marker not present in the panel (36, 37), it characterizes additional markers from the different panels using the UMAP coordinates generated with the information of backbone markers common to all panels. It also allowed us to investigate the expression of non-backbone markers across FlowSOM clusters and a pseudotime trajectory again defined by the backbone marker information. Notably, our strategy can likely be applied to more panels with more markers and in multiple biological contexts.

The analysis performed within the putative total Treg significantly increased the resolution, granting the identification of relevant clusters present in thymic tissue from donors of different ages. The inclusion of thymocytes only expressing CD25 or FOXP3 was decided based on our previous data on human Treg development (15, 38) and to fit with the FOXP3-first and CD25-first precursors described in mice (5, 12). However, we were aware of possible contamination by conventional thymocytes, as both markers can be upregulated following activation (39). Our study does not attempt to provide definitive answers into the relationships between cells classified in different clusters, but the marker information and trajectory inference analysis provide significant contributions. We understand the limitations in trajectory inference analysis, and possible biases introduced when choosing start and endpoints. However, our choices were founded on solid knowledge on thymic development and our analysis was intended to generate hypothesis to be further probed. Thymocytes expressing only one of these Treg-lineage markers were found along the T cell development, namely in DP clusters expressing higher levels of CD1a and in mature clusters defined by high levels of CD45RA in CD4SP and CD8SP. It is, therefore, tempting to consider that the CD1a expressing DP thymocytes correspond to the two alternate pathways previously suggested in line with an earlier Treg commitment in the human thymus (5, 6, 12, 13, 18, 40). However, the current algorithms did not retrieve pseudotime trajectories initiating in the two populations, a limitation expected to be solved soon given the rapid development of applications and the expanding number of markers in the panels. It is also important to contextualize our data to the recent studies using CITE-seq and single-cell transcriptomics of human thymocytes, which either do not detect or consider a DP Treg-committed population (25, 33), detect but do not discuss its implications (7) or detect and discuss, leaving open Treg commitment at more than one-time point across pseudotime (6).

Of particular interest in our analysis are CD8SP Treg clusters, as, possibly due to their rarity, these cells are not considered in trajectory inference studies with human thymocytes (6, 28, 33), and are found annotated only in the most recent human thymus atlas (8). We investigated two different CD8SP endpoints, clearly diverging halfway in the trajectory. One of the branches expressed CD25 and negligible levels of FOXP3 and CTLA-4 throughout the trajectory and includes a fraction of the CD25-only DP cluster (cluster 6) that failed to map to other trajectories. This branch is likely to include non-Treg cells. In contrast, the other CD8SP trajectory expressed FOXP3 and CD103. CD103, an integrin that dimerizes to integrin β7 and binds to E-cadherin (41), is already expressed at the DP stage, apparently marking CD8 Treg-lineage choice, as previously suggested (18). Thus, CD103 represents an relevant marker to sort and functionally investigate CD8 Tregs in the human thymus.

Interestingly, although we were able to identify mature (CD45RA^pos^) CD4SP Tregs expressing either CD25 or FOXP3, it was not possible to identify a marker or marker combination clearly separating these trajectories, possibly reflecting greater fluidity in CD4 Treg commitment and the variable times of induction found for CD4SP Tregs (6).

Finally, the trajectory leading to CXCR3^pos^ CD4SP presents significant overlap to both other lineages, however, it appears to represent a different fate, with a distinctive phenotype characterized by the highest expression of the *bona fide* Treg markers, such as FOXP3, CD25 and CTLA-4, as well as the highest expression of CXCR3, T-bet and ICAM-1 and lowest levels of TCF-1. This phenotype is in concordance with the phenotype of a population of cells described as recirculating in studies from both mice and humans (7, 8, 14, 33, 42). A recent study using a recently described novel trajectory inference methodology named *tviblindi* and different datasets, also identified a population with the same phenotype at the end of a trajectory (28). Nevertheless, it must be noted that these cells could also be thymic-resident and that the expression of CD45RO would also agree with an intermediate developmental stage. In line with this possibility is the observation of CD8α, CD8β, and CD1a expression in this cluster, albeit at low levels. Although CD8 reexpression has been associated with activated CD4 T cells in tissues (43), the expression of CD1a is intriguing and could be compatible with the coexistence within this population of cells at different developmental stages. Interestingly, in our comparative analysis of samples from children, adolescents, and older individuals, the CXCR3-expressing population subdivides into two clusters, differing in the expression of CD8 and CD1a. The cluster expressing CD8 and CD1a was almost absent in the samples from older individuals, as are clusters corresponding to most immature developmental stages, suggesting that it may represent immaturity rather than an activated state. In contrast to the lack of increase of CXCR3^pos^ CD4SP Tregs in the older thymi, we do observe a marked expansion in clusters featuring an activated or possibly recirculating phenotype, namely expressing CD25, CD127 and negligible levels of FOXP3. These clusters may represent Tconvs and were rare in the thymic tissue of children and adolescents. We were careful during tissue processing to avoid any blood-derived contamination, but a possible relation of these cells with the adipose tissue observed in the thymic samples from older individuals cannot be excluded and remains to be investigated. The function of the CD25-only populations in the human thymus should be investigated in future studies. Our results support a function far beyond being precursors of *bona fide* FOXP3 Tregs. Additionally, we showed that the expression of CD25 not accompanied by FOXP3 was associated with distinct phenotypes in CD8SP and CD4SP. It would be important to define if the CD25-only populations have regulatory properties or if they play a role in the maturation of conventional thymocytes and may impose any functional imprint.

In conclusion, we investigated human thymic Treg heterogeneity using a spectral flow cytometry approach, that can be applied to other population-focused studies. The simplicity of the approach takes advantage of the many channels offered by spectral flow cytometry and of existing unsupervised analysis algorithms to visualize the expression of markers, projected into UMAPs and FlowSOM clusters defined by a backbone. Based on the acquisition of large numbers of thymocytes, we subcluster putative total Tregs and identify Treg subpopulations, likely to represent relevant alternate paths of development for CD4 and CD8 Tregs, in addition to possible thymic resident locally activated or recirculating populations. While the hypotheses generated by our analysis need fate-mapping studies with human thymocytes for validation, our flow cytometry data may provide handles for the design of these experiments.

## Data Availability

The raw data supporting the conclusions of this article will be made available by the authors, without undue reservation.
