# Supplementary material for: A backbone-based flow cytometry approach to decipher regulatory T cell trajectories in the human thymus
Source: Front Immunol. 2025 Mar 3;16:1553535. doi: 10.3389/fimmu.2025.1553535 (PMC11911493; doi:10.3389/fimmu.2025.1553535)
Supplement: Supplementary file 1 [file DataSheet1.pdf]

A backbone-based flow cytometry approach to decipher regulatory T cell trajectories in the human thymus

Beatriz Moleirinho[1,2†], Margarida Paulo-Pedro[1,2†], Nicole C. Martins[1,2†], Emily Jelagat[1,2], Eller Conti[1,2], Tiago R. Velho[2,3], Miguel Abecasis[4], Rui Anjos[4], Afonso R.M. Almeida[1,2§] and Ana E. Sousa[1,2§]

[1] GIMM - Gulbenkian Institute for Molecular Medicine, Lisbon, Portugal
[2] Faculdade de Medicina, Universidade de Lisboa, Lisbon, Portugal
[3] Hospital de Santa Maria, Unidade Local de Saúde de Santa Maria, Lisbon, Portugal
[4] Hospital de Santa Cruz, Unidade Local de Saúde de Lisboa Ocidental, Carnaxide, Portugal

[†] These authors contributed equally to this work and shared first authorship
[§] These authors share last authorship

Supplementary Material

1 List of Supplementary Materials

Supplementary Figure 1. ***Gating strategy for all thymocytes and Tregs*** (Related to Figure 1A).
Supplementary Figure 2. ***Analysis of total thymocytes using UMAPs*** (Related to Figure 1A).
Supplementary Figure 3. ***Comparison of backbone marker expression within each panel.*** (Related to Figure 1A and 1B).
Supplementary Figure 4. ***FlowSOM clustering of human thymic Tregs.*** (Related to Figure 1C).
Supplementary Figure 5. ***Cluster distribution variation within thymic Tregs.*** (Related to Figure 1C).
Supplementary Figure 6. ***Non-backbone marker expression in thymic Tregs.*** (Related to Figure 2A and 2B).
Supplementary Figure 7. ***Analysis of cytokine production by total thymocytes.***
Supplementary Figure 8. ***Illustrative analysis of the expression of CTLA4, CXCR3 and CD103 in total putative Tregs using manual analysis.***
Supplementary Figure 9. ***Non-backbone marker expression projection in diffusion map of human thymic Tregs.*** (Related to Figure 3A).
Supplementary Figure 10. ***Illustrative analyses of thymocytes from elder subjects.*** (Related to Figure 5A).
Supplementary Figure 11. ***UMAPs of total thymocytes and Tregs including elder subjects.*** (Related to Figure 5A).
Supplementary Figure 12. ***FlowSOM clustering of data including elder subjects.*** (Related to Figure 5A).

Supplementary Table 1. List of thymus samples used in the study
Supplementary Table 2. List of antibodies used in flow cytometry panels
Supplementary Table 3. List of software and algorithms used

2 Supplementary Figures and Tables

2.1 Supplementary Figures

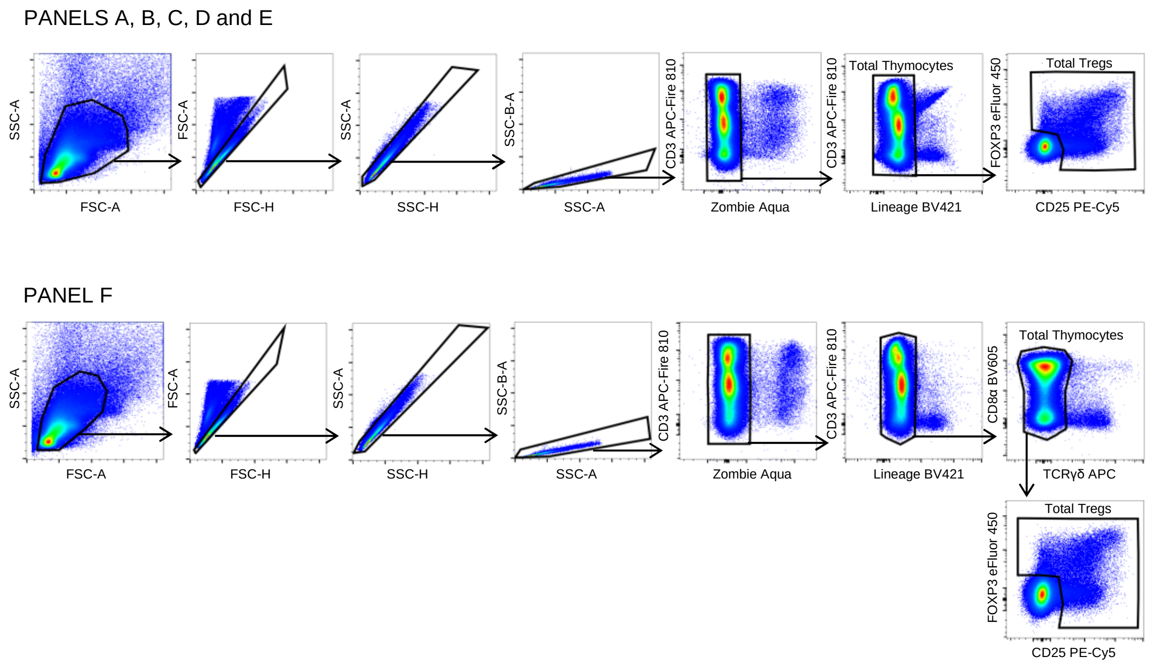

Supplementary Figure 1. ***Gating strategy for all thymocytes and regulatory T cells (Tregs).*** Illustrative gating strategies applied to the FCS files used in the initial step of the R-studio pipeline (in grey in Figure 1A); (Top) panels A, B, C, D and E where the anti-TCRγδ antibody was included in the lineage channel; (Bottom) panel F where the anti-TCRγδ antibody was included in a distinct channel; γδ thymocytes were always excluded from the total thymocytes' analysis; other lineage markers were: CD11c, CD14, CD16, CD19 and CD123. (Related to Figure 1A).

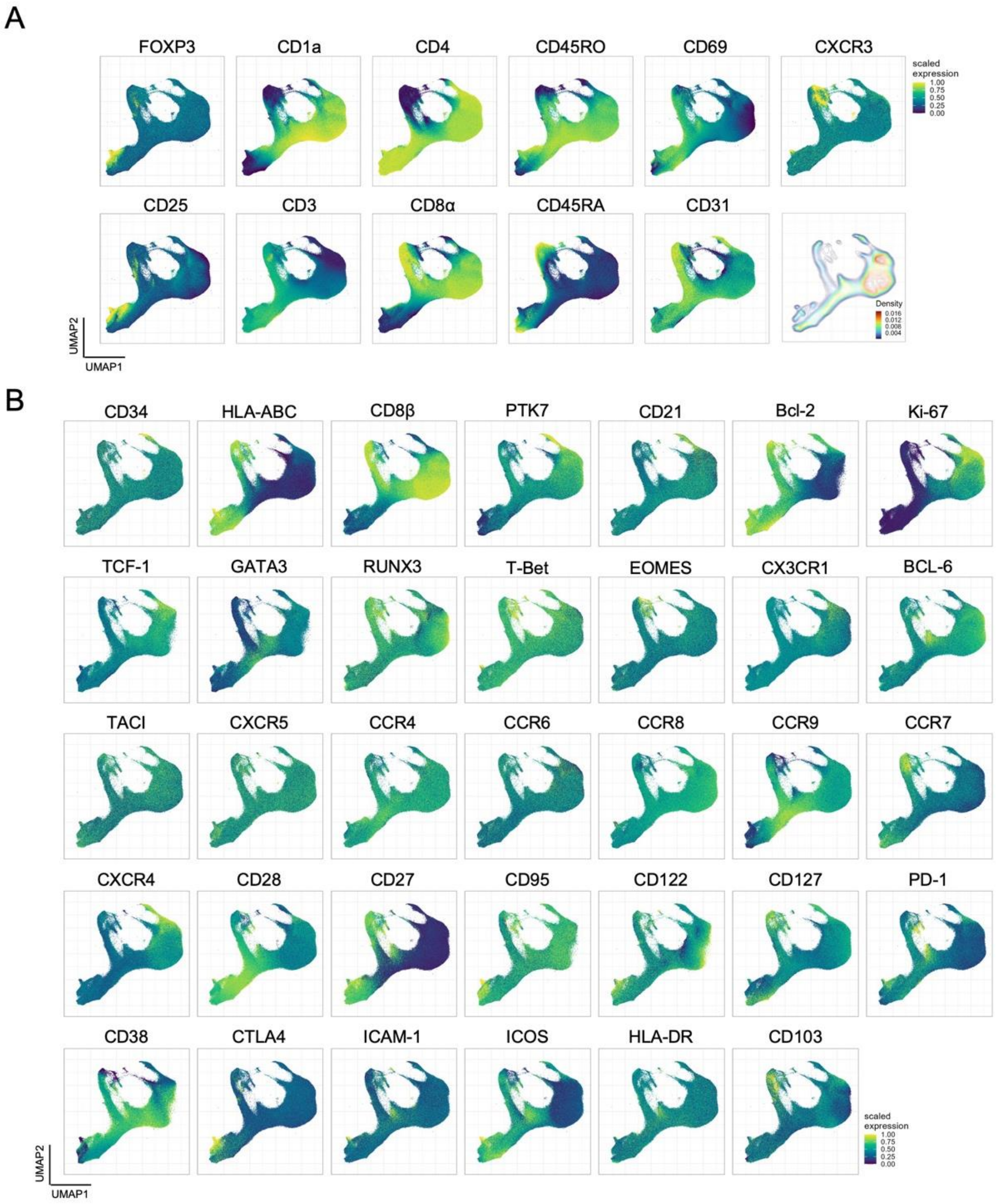

Supplementary Figure 2. ***Analysis of total thymocytes*****.** **(A).** UMAP of total thymocytes showing scaled expression of backbone markers and density plot with distribution of events (*n*=5.630.522) (bottom right), upon application of the same R-Studio pipeline described in Figure 1A; expression of the backbone markers allows the identification of UMAP regions corresponding to most important thymocytes subpopulations (DN, DP, CD4SP, CD8SP, CD4 Treg). **(B).** Scaled expression of selected markers from panels A to F projected onto the same UMAP. Total thymocytes were gated excluding other lineage markers and γδ thymocytes. (Related to Figure 1A).

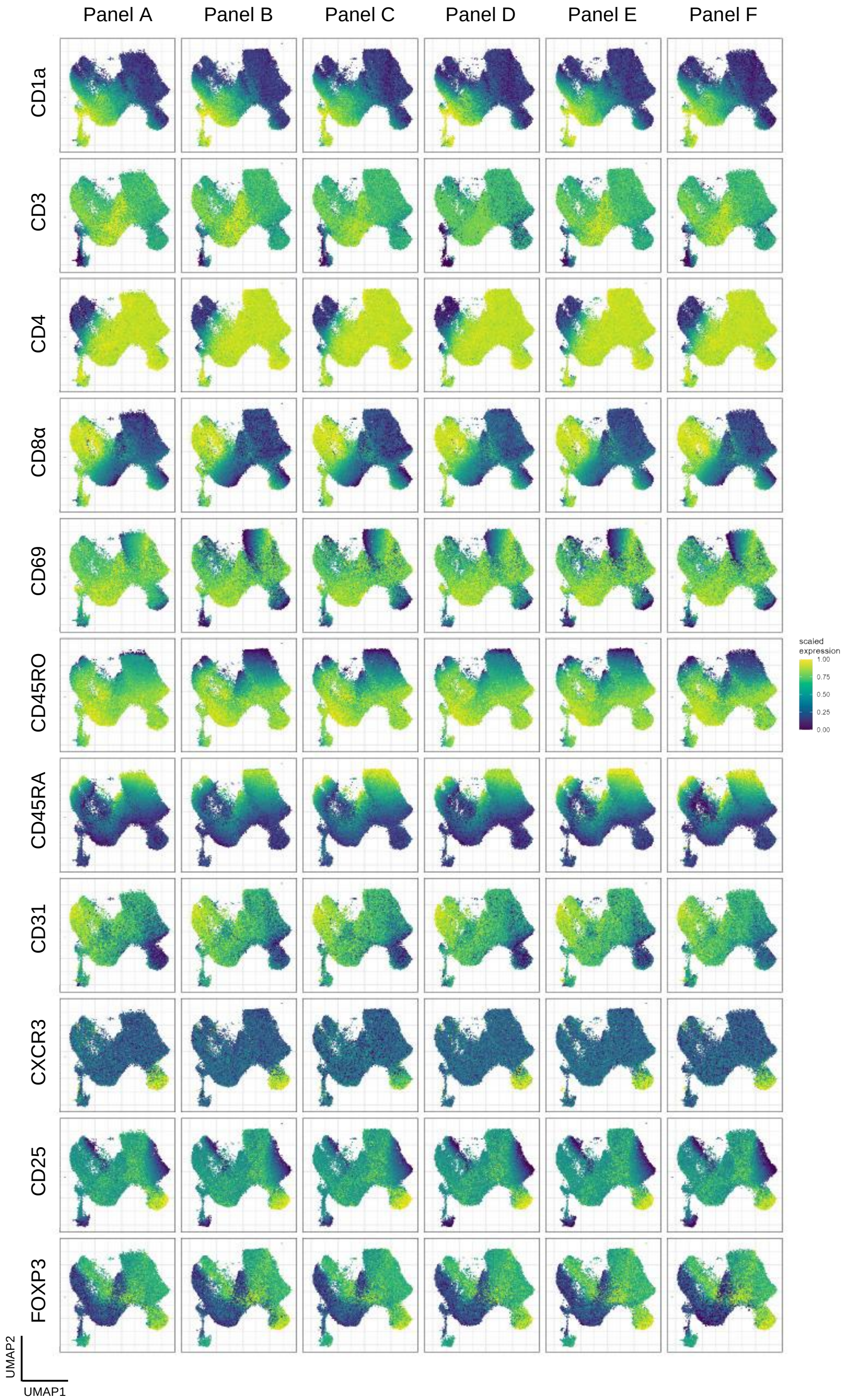

Supplementary Figure 3. ***Comparison of backbone markers' expression within each panel.*** UMAPs of total Tregs from each panel (A-F) showing the expression of the backbone markers; notably equivalent profiles independent of the panel of origin were displayed (Related to Figure 1A and 1B).

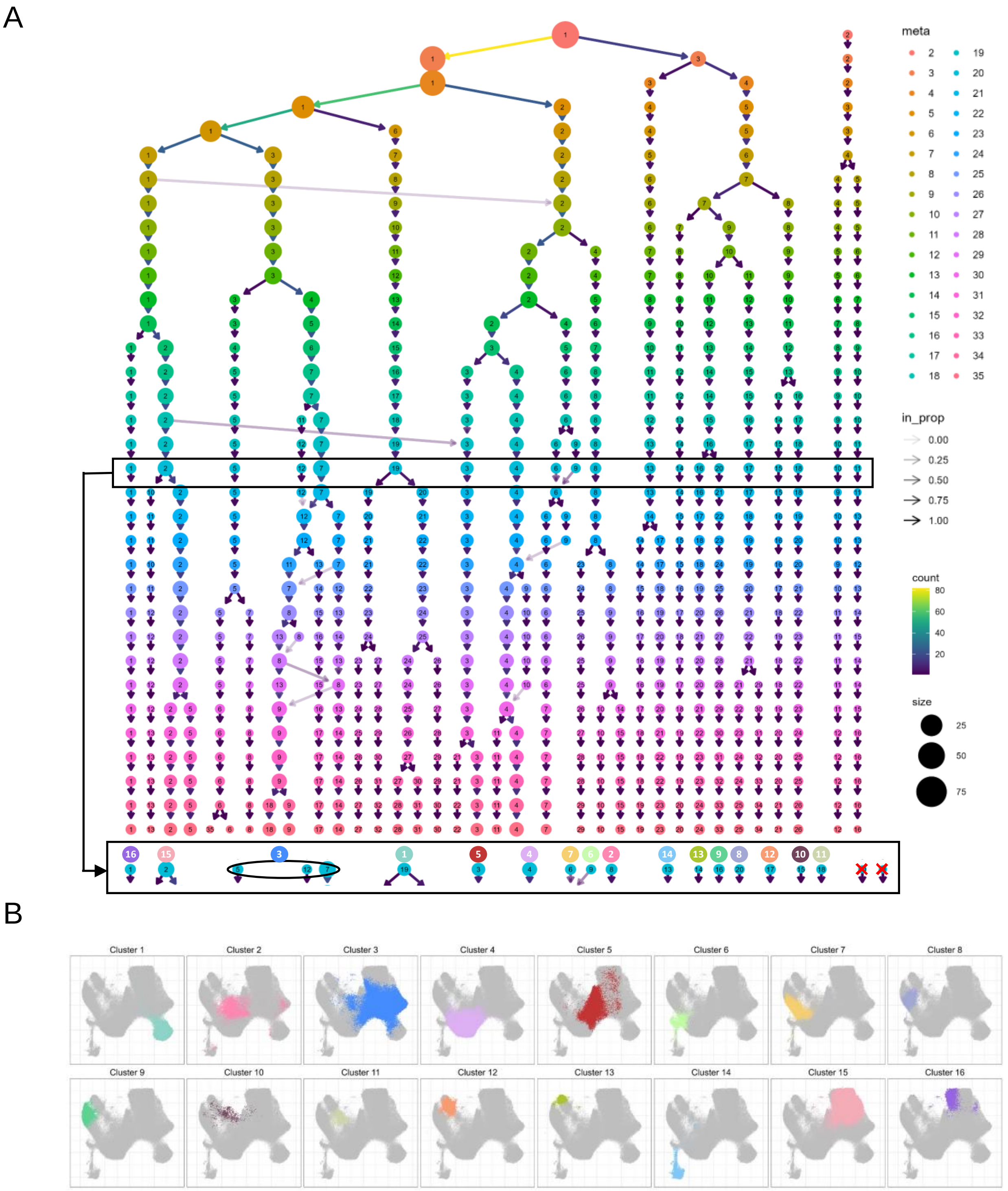

Supplementary Figure 4. ***FlowSOM clustering of human thymic Tregs*****. (A).** Clustree visualization of the resulting clusters in Tregs using FlowSOM at different meta parameters, denoted by row; the number of circles in each row represents the number of clusters found, with the circle size representing the number of cells; rectangles highlight the meta chosen with 20 clusters (top) and the clusters merged, removed and renamed (bottom). **(B).** UMAP plots of total Tregs showing each of the 16 clusters. (Related to Figure 1C).

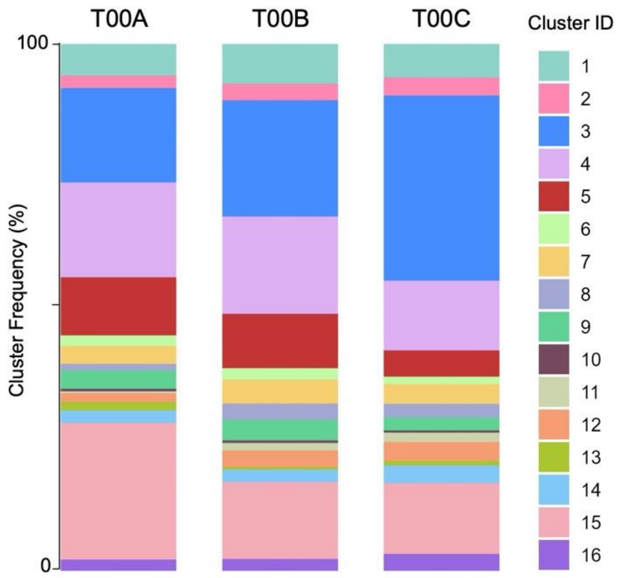

Supplementary Figure 5. ***Cluster distribution variation within thymic Tregs.*** Stacked bar graphs present the proportion of the 16 clusters in the three different human thymi included in the analysis. (Related to Figure 1C).

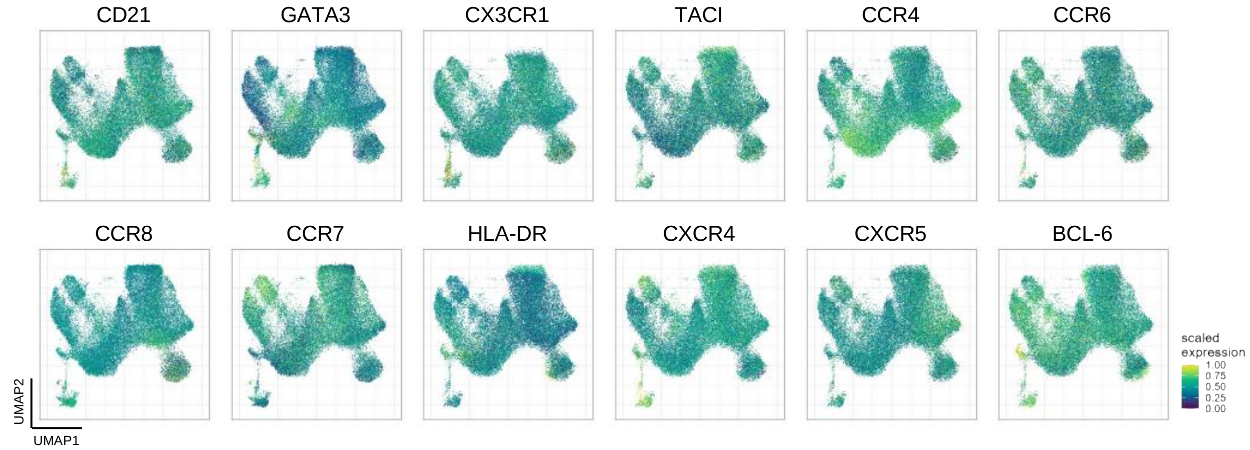

Supplementary Figure 6. ***Non-backbone marker expression in thymic Tregs.*** Scaled expression of other markers from different panels not included in the main Figure 2, projected onto the UMAP generated from backbone marker expression. (Related to Figure 2A and 2B).

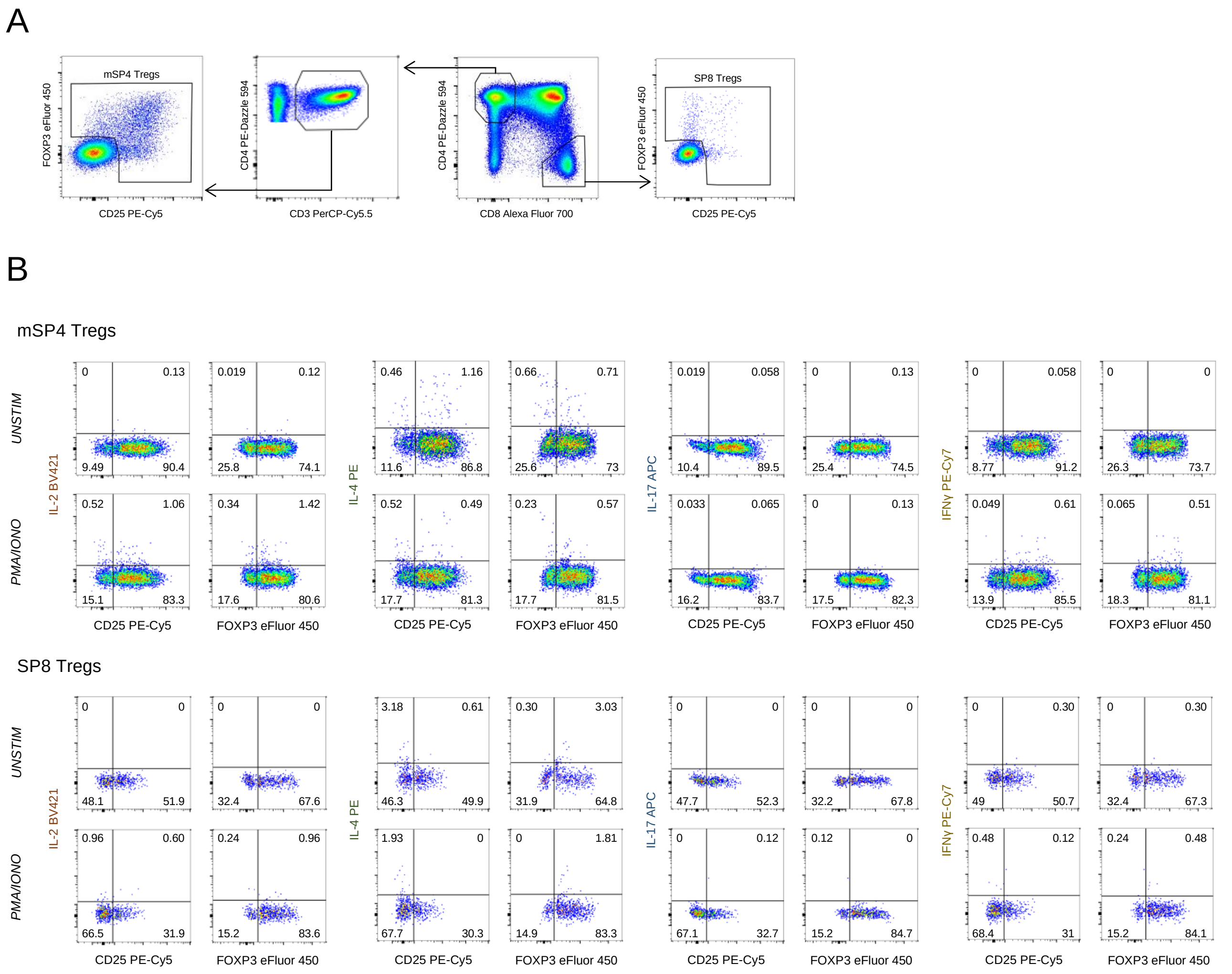

Supplementary Figure 7. ***Analysis of cytokine production by total thymocytes.*** **(A)** Illustrative gating strategy for analysis of mature single positive CD4 Tregs and single positive CD8 Tregs. **(B)** Illustrative dot-plots showing the analysis of cytokine production (IL-2, IL-4, IL-17, and IFNγ, from left to right) without stimulation and upon PMA/Ionomycin stimulation, in CD4SP Tregs (mSP4, top) and CD8SP Tregs (SP8, bottom).

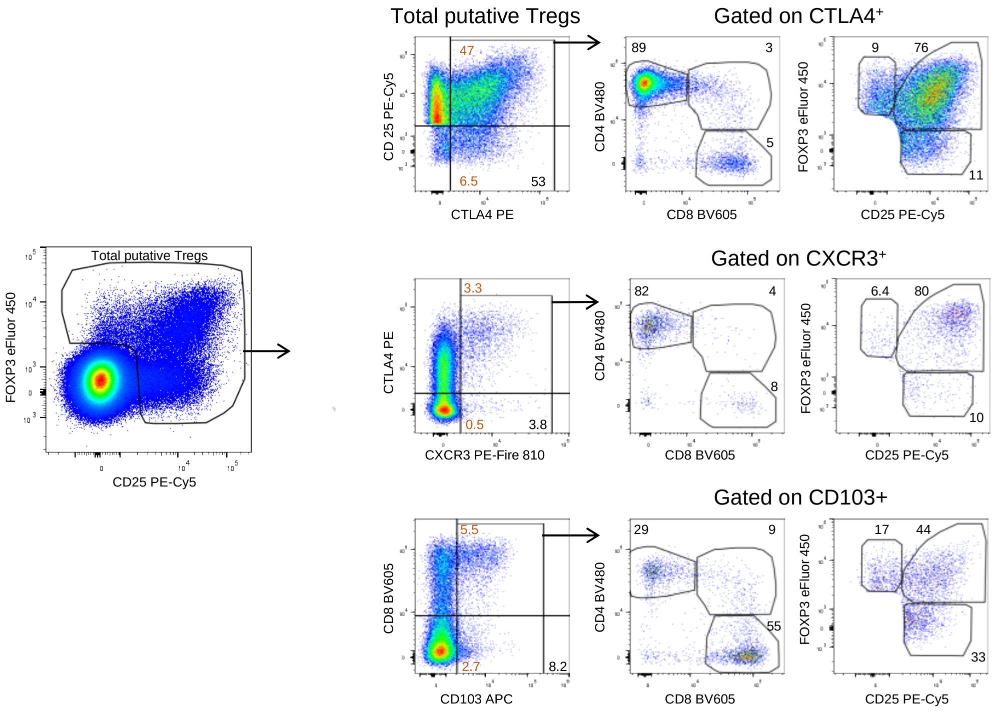

Supplementary Figure 8. ***Illustrative analysis of the expression of CTLA4, CXCR3 and CD103 in total putative Tregs using manual analysis.*** Total putative Tregs of a representative pediatric thymus sample were gated (left) and the expression of CTLA4 (top), CXCR3 (middle) and CD103 (bottom) is shown. Subsequently, CTLA4+, CXCR3+ and CD103+ cells were gated and CD4/CD8 and FOXP3/CD25 dot plots are shown (right). Numbers in plots are frequencies inside the gates, frequencies in respective quadrants are shown in orange.

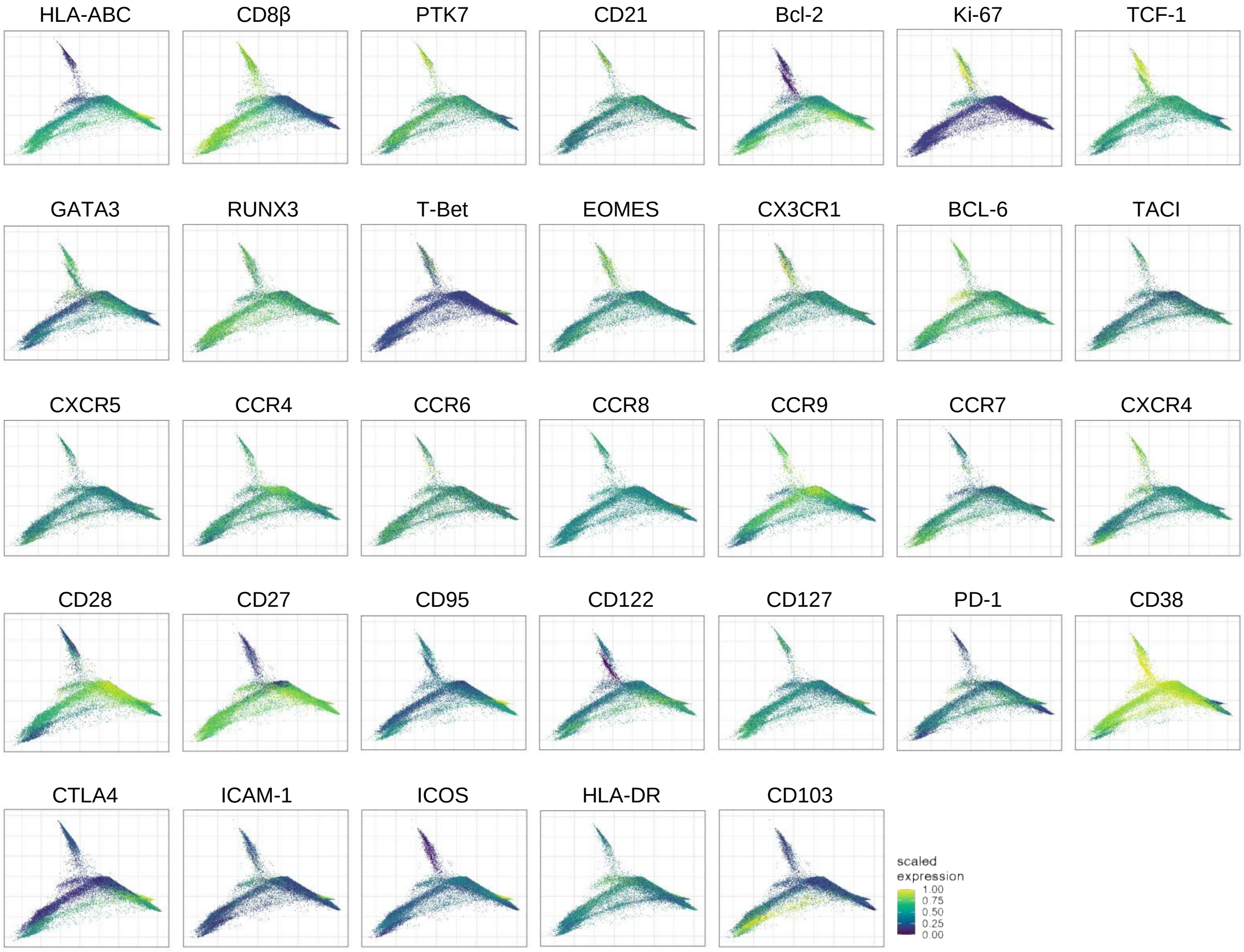

Supplementary Figure 9. ***Non-backbone marker expression projection in diffusion map of human thymic Tregs.*** Scaled expressions of non-backbone markers from panels A-F, projected onto the diffusion map generated from backbone markers expression and shown in Figure 3. (Related to Figure 3A).

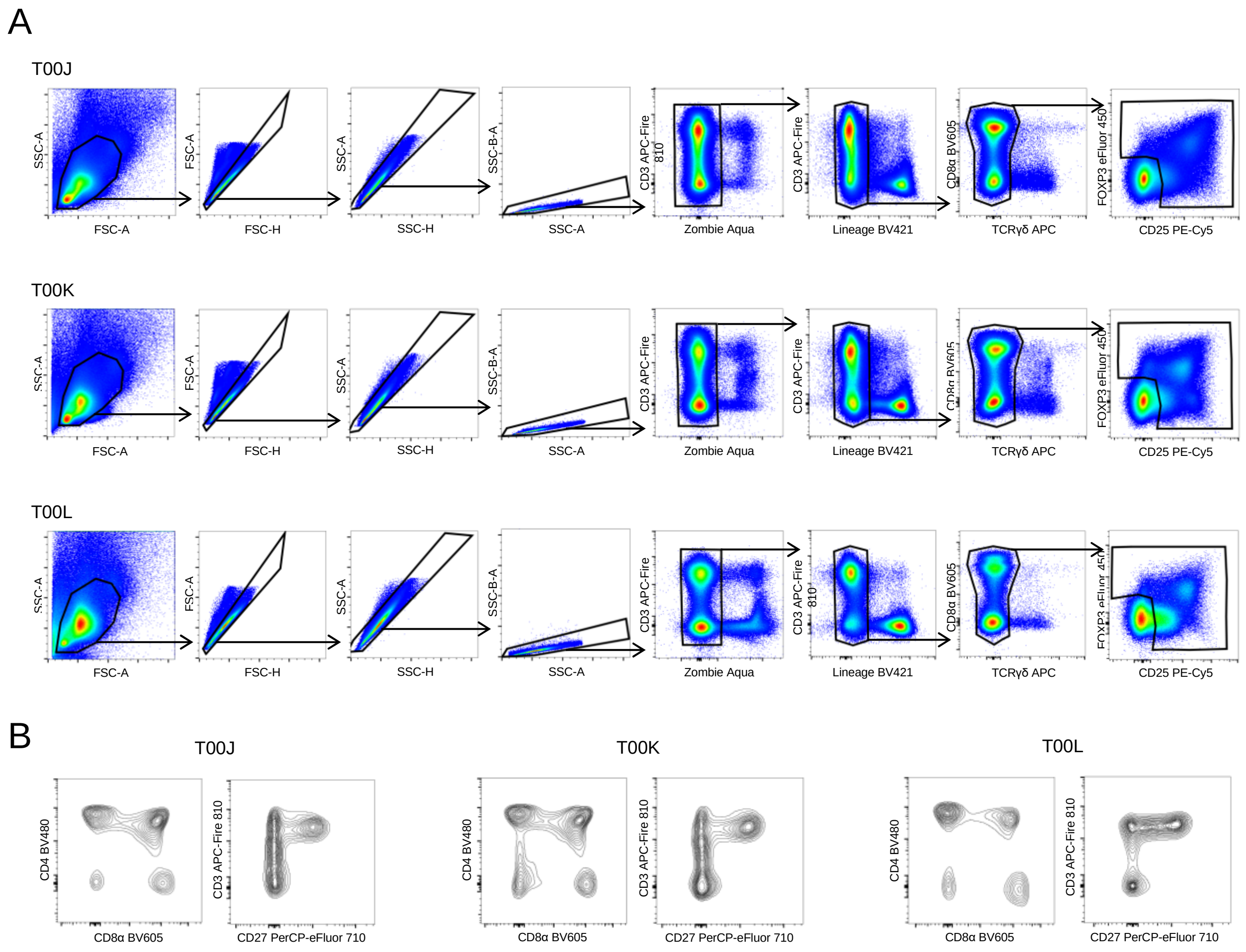

Supplementary Figure 10. ***Illustrative analyses of thymocytes from elderly subjects*****. (A).** Gating strategy for total thymocytes from each individual with ≥74-year-old included in data shown in Figure 5; as Panel F was used, γδ-T cells were excluded before the export of total Treg events. **(B)**. Contour plots show the analysis of CD4 vs CD8 and CD3 vs CD27 expression in the same samples as in **(A)**. (Related to Figure 5A).

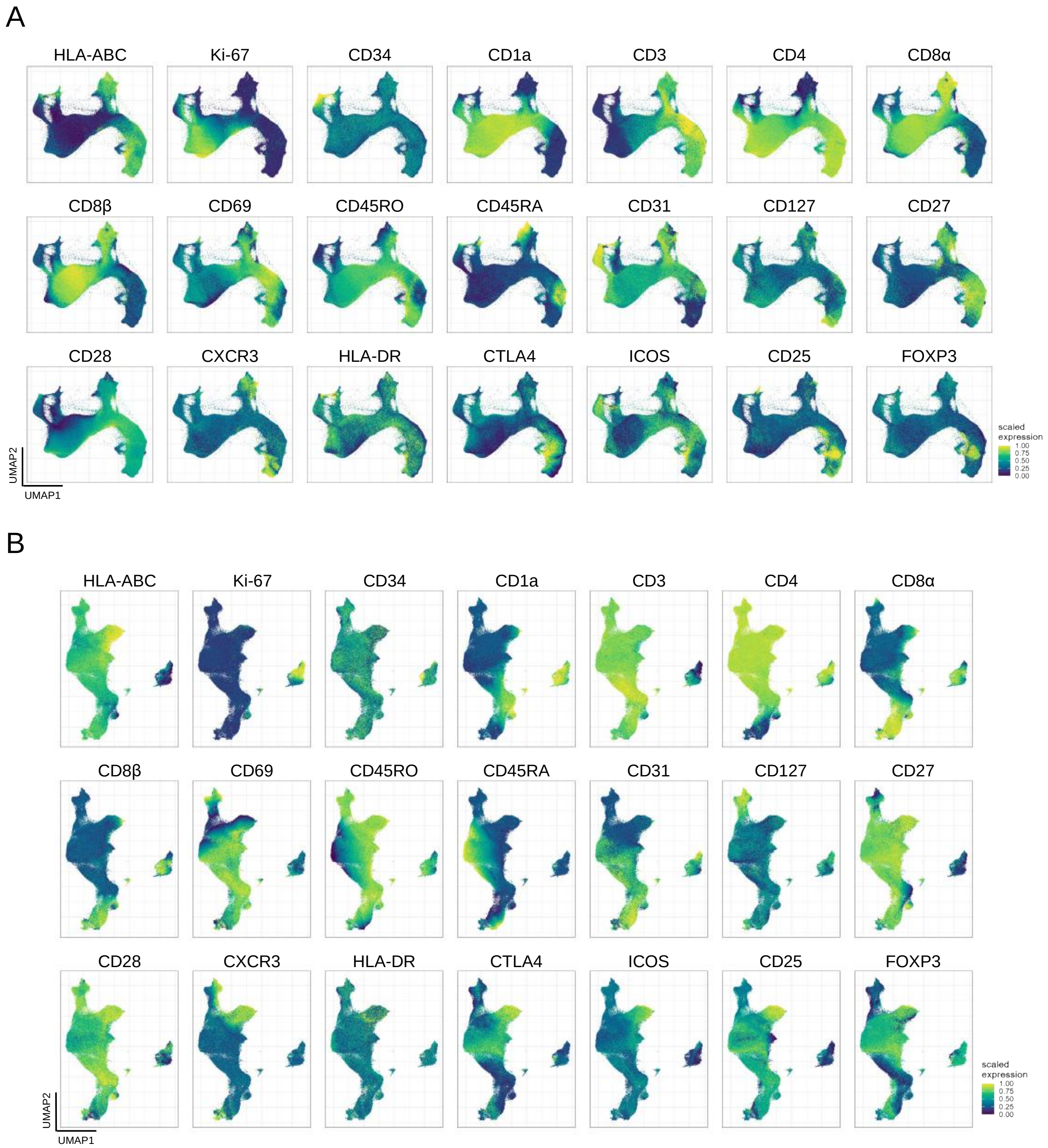

Supplementary Figure 11. ***UMAPs of total thymocytes and Tregs including elder subjects*****. (A).** Scaled expressions of all markers from Panel F in total thymocytes (*n*=3.973.944). (**B**). Scaled expression of markers from Panel F in total Tregs (*n*=241.574). Concatenated data from all thymi included in Figure 5 are shown. (Related to Figure 5A).

A

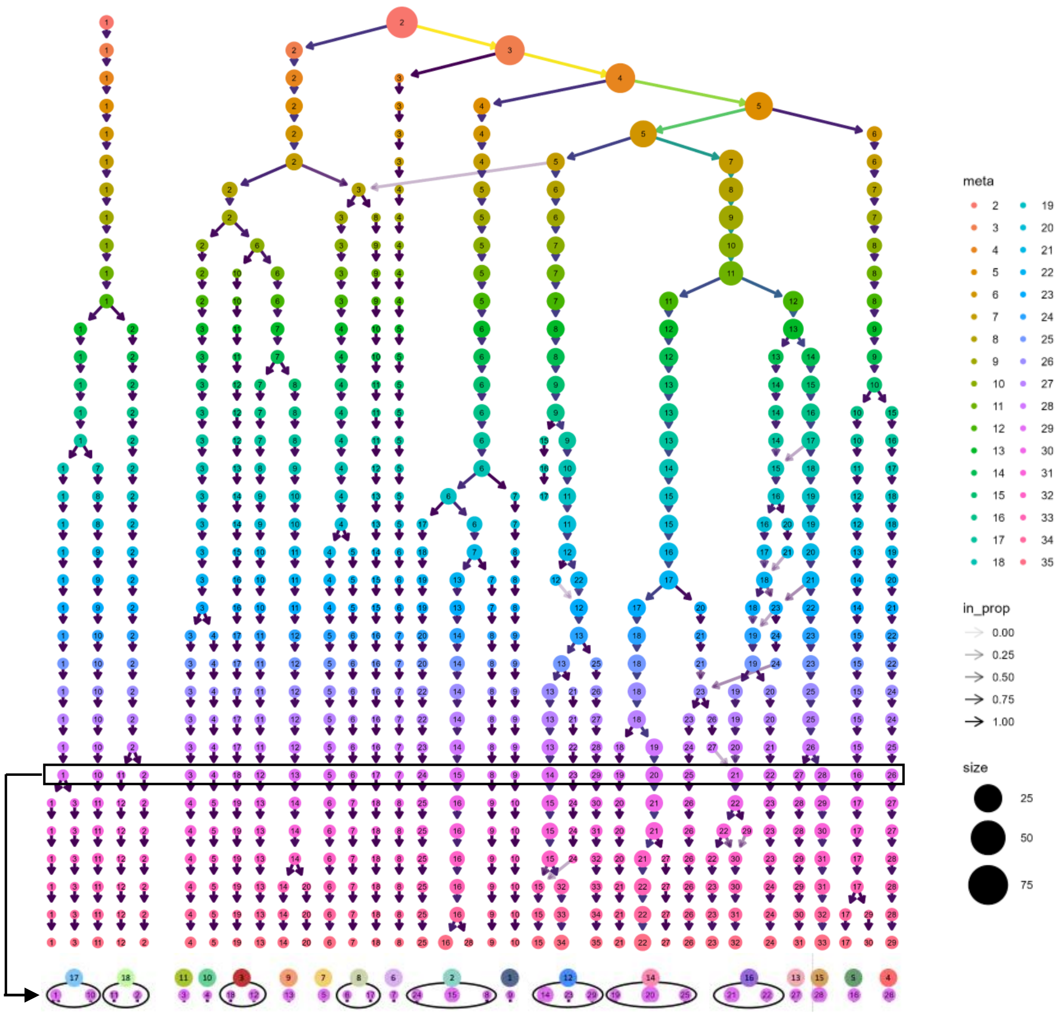

B

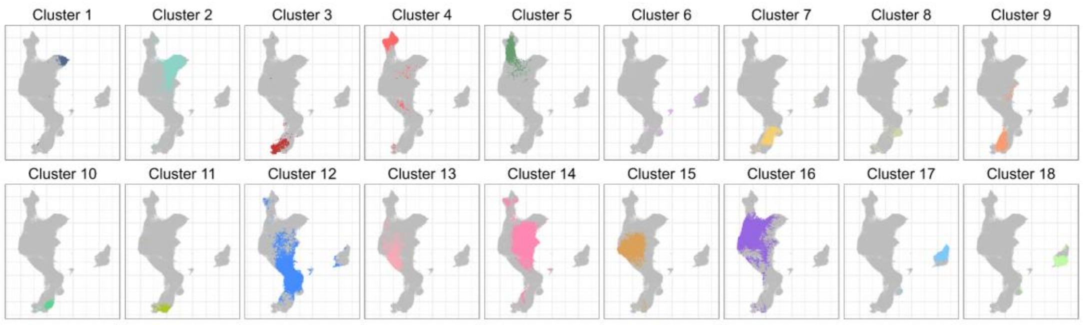

Supplementary Figure 12. ***FlowSOM clustering of data including elderly subjects*****. (A).** Clustree visualization of the resulting clusters in total Tregs using FlowSOM at different meta parameters, denoted by row; the number of circles in each row represents the number of clusters found, with the circle size representing the number of cells; rectangles highlight the meta chosen with 20 clusters (top) and the clusters merged, removed and renamed (bottom). **(B)**. UMAP plots show the individual projections of each of the 18 final clusters. (Related to Figure 5B).

2.2 Supplementary Tables

Supplementary Table 1. List of thymus samples used in the study.

Thymus	Age (Years)	Gender at Birth	Surgery Motive	Figures
T00A	0.75 (8 months)	Male	Cardiac Reconstructive Surgery	1-5
T00B	0.75	Male	Cardiac Reconstructive Surgery	1-5
T00C	0.75	Male	Cardiac Reconstructive Surgery	1-5
T00D	0.6 (7 months)	Female	Cardiac Reconstructive Surgery	5
T00E	1	Female	Cardiac Reconstructive Surgery	5
T00F	6	Female	Cardiac Reconstructive Surgery	5
T00G	5	Female	Cardiac Reconstructive Surgery	5
T00H	15	Male	Cardiac Reconstructive Surgery	5
T00I	8	Male	Cardiac Reconstructive Surgery	5
T00J	75	Male	Aortic Stenosis	5
T00K	77	Male	Aortic Stenosis	5
T00L	74	Male	Coronary Artery Disease	5

Supplementary Table 2. List of antibodies used in flow cytometry panels

Antigen	Clone	Fluorophore	Source	Dilution	Staining	Panel
Bcl-2	124	FITC	Dako	1/50	Intracellular	C
BCL6	K112-91	Alexa Fluor 647	BD	1/60	Intracellular	D
CCR4	1G1	BV750	BD	1/86	Surface	B, D, E
CCR6	R6H1	PE-Cy7	eBioscience	1/50	Surface	B, D, E
CCR7	150503	FITC	R&D Systems	1/15	Surface	E
CCR8	L263G8	APC	BioLegend	1.5/100	Surface	E
CCR9	LO53E8	PE	BioLegend	1/45	Surface	E
CD103	Ber-ACT8	APC	BioLegend	1/100	Surface	A, B
CD11c	3.9	BV421	BioLegend	1/150	Surface	Lineage
CD122 (IL-2Rβ)	TU22	APC	BioLegend	1.5/100	Surface	C
CD123	6H6	BV421	BioLegend	1/150	Surface	Lineage
CD127	A019D5	Alexa Fluor 700	BioLegend	3.5/150	Surface	B
CD127	HIL-7R-M21	BV711	BD	1/75	Surface	A, C, D, E, F
CD14	HCD14	BV421	BioLegend	1/150	Surface	Lineage
CD16	B73.1	BV421	BioLegend	1/150	Surface	Lineage
CD184 (CXCR4)	12G5	APC	eBioscience	1/75	Surface	D
CD185 (CXCR5)	J252D4	PE-Dazzle594	BioLegend	1/100	Surface	D
CD19	HIB19	BV421	BioLegend	1/150	Surface	Lineage
CD1a	HI149	APC-Fire 750	BioLegend	1/100	Surface	Backbone
CD21	B-ly4	BV711	BD	1/200	Surface	B
CD25	BC96	PE-Cy5	BioLegend	1/125	Surface	Backbone, Cytokines
CD267 (TACI)	11H3	PE	eBioscience	1/30	Surface	D
CD27	O323	FITC	Invitrogen	1/75	Surface	A
CD27	O323	PerCP-eFluor 710	eBioscience	1/50	Surface	B,C,D,E,F
CD278 (ICOS)	C398.4A	Alexa Fluor 700	BioLegend	1/50	Surface	A,C,D,E,F
CD279 (PD-1)	MIH4	FITC	Invitrogen	1/50	Surface	D
CD28	CD28.2	PE-Cy7	eBioscience	1/50	Surface	F
CD3	OKT3	PerCP-Cy5.5	eBioscience	1/200	Surface	Cytokines
CD3	SK7	APC-Fire 810	BioLegend	1/175	Surface	Backbone
CD31	WM59	BV785	BioLegend	1/250	Surface	Backbone
CD34	581	PE-Dazzle594	BioLegend	1/150	Surface	F

Supplementary Table 2. List of antibodies used in flow cytometry panels *(continued)*

Antigen	Clone	Fluorophore	Source	Dilution	Staining	Panel
CD38	S17015A	PE-Fire 700	BioLegend	1/150	Surface	C
CD4	RPA-T4	PE-Dazzle594	BioLegend	1/200	Surface	Cytokines
CD4	SK3	BV480	BD	1/150	Surface	Backbone
CD45RA	HI100	BV510	BD	1/150	Surface	Cytokines
CD45RA	HI-100	BV650	BioLegend	1/100	Surface	Backbone
CD45RO	UCHL1	BV570	BioLegend	1/100	Surface	Backbone
CD54 (ICAM-1)	HA58	PE-Dazzle594	BioLegend	1/100	Surface	A,B,E
CD69	FN50	BV785	BioLegend	1/300	Surface	Cytokines
CD69	FN50	Spark NIR 685	BioLegend	1/200	Surface	Backbone
CD8α	RPA-T8	BV605	BioLegend	1/200	Surface	Backbone
CD8α	SK1	Alexa Fluor 700	BioLegend	1/150	Surface	Cytokines
CD8β	SIDI8BEE	eFluor 660	eBioscience	1/100	Surface	F
CD95	DX2	PE-Cy7	eBioscience	1/150	Surface	C
CTLA4	BNI3	PE	BD	1/100	Intracellular	F
CX3CR1	HA58	PerCP-eFluor 710	BioLegend	1/100	Surface	A
CXCR3	G025H7	PE-Fire 810	BioLegend	1/200<br>1/300	Surface	Backbone,<br>Cytokines
EOMES	WD1928	PE	eBioscience	1/75	Intracellular	A
FOXP3	PCH101	eFluor 450	eBioscience	1/100	Intracellular	Backbone,<br>Cytokines
GATA3	TWAJ	PE-eFluor 610	Invitrogen	1/25	Intracellular	C
HLA-ABC	WA/32	FITC	eBioscience	1/50	Surface	B,F
HLA-DR	L243	Spark Violet 538	BioLegend	1/100	Surface	A,D,F
IFNγ	4S.B3	PE-Cy7	Invitrogen	1/200	Intracellular	Cytokines
IL-17	eBio64DEC 17	APC	Invitrogen	1/30	Intracellular	Cytokines
IL-2	MQ1-17H12	BV421	BioLegend	1/150	Intracellular	Cytokines
IL-4	8D4-8	PE	Invitrogen	1/50	Intracellular	Cytokines
Ki-67	B56	PerCP-Cy5.5	Invitrogen	1/300	Intracellular	A,B,C,D,<br>E,F
Live/Dead	Not applicable	NIR	Invitrogen	1/200	Surface	Cytokines
Live/Dead	Not applicable	Zombie Aqua	BioLegend	1/500	Surface	A,B,C,D,<br>E,F
RUNX3	R3-5G4	eFluor 660	eBioscience	1/1200	Intracellular	A
T-Bet	eBio4B10	PE-Cy7	eBioscience	1/200	Intracellular	A

Supplementary Table 2. List of antibodies used in flow cytometry panels *(continued)*

Antigen	Clone	Fluorophore	Source	Dilution	Staining	Panel
TCF-1	7F11A10	PE	BioLegend	1/100	Intracellular	C
TCRγδ	B1	APC	BioLegend	1/50	Surface	F (Lineage)
TCRγδ	B1	BV421	BioLegend	1/50	Surface	A,B,C,D, E (Lineage)
TCRγδ	B1.1	FITC	eBioscience	1/30	Surface	Cytokines

Supplementary Table 3. List of software and algorithms used

Software/Algorithm	Source	Identifier
SpectroFlo v3.2.1	Cytek Biosciences	https://cytekbio.com/pages/spectro-flo
Prism v8.4.3	GraphPad	https://www.graphpad.com/scientific-software/prism/
R v4.3.3	R Core Team	https://cran.r-project.org/mirrors.html
FCS Express v7.18	De Novo Software	https://denovosoftware.com/
flowCore v2.14.2	Hahne et al. (1)	https://github.com/RGLab/flowCore
FlowVS v1.34.0	Azad et al. (2)	https://github.com/azadcse/flowVS
flowAI v1.32.0	Monaco et al. (3)	https://github.com/giannimonaco/flowAI
FlowStats v4.14.1	Hahne et al. (4)	https://github.com/RGLab/flowStats
CATALYST v1.26.1	Crowell et al. (5)	https://github.com/HelenaLC/CATALYST
uwot v0.1.16	Melville et al. (6)	https://github.com/jlmelville/uwot
clustree v0.5.1	Zappia et al. (7)	https://github.com/lazappi/clustree
ggplot2 v3.5.0	Wickham et al. (7)	https://cloud.r-project.org/web/packages/rgeos/index.html

References of Supplementary Materials:

1. Hahne F, LeMeur N, Brinkman RR, Ellis B, Haaland P, Sarkar D, Spidlen J, Strain E, Gentleman R. flowCore: a Bioconductor package for high throughput flow cytometry. *BMC Bioinformatics* (2009) 10:106. doi: 10.1186/1471-2105-10-106

2. Azad A, Rajwa B, Pothen A. flowVS: channel-specific variance stabilization in flow cytometry. *BMC Bioinformatics* (2016) 17:291. doi: 10.1186/s12859-016-1083-9

3. Monaco G, Chen H, Poidinger M, Chen J, De Magalhães JP, Larbi A. flowAI: automatic and interactive anomaly discerning tools for flow cytometry data. *Bioinformatics* (2016) 32:2473–2480. doi: 10.1093/bioinformatics/btw191

4. Hahne F, Gopalakrishnan N, Khodabakhshi A, Wong C, Lee K. flowStats: Statistical methods for the analysis of flow cytometry data. *R package version 4180* http://www.github.com/RGLab/flowStats

5. Nowicka M, Krieg C, Crowell H, Weber L, Hartmann F, Guglietta S, Becher B, Levesque M, Robinson M. CyTOF workflow: differential discovery in high-throughput high-dimensional cytometry datasets [version 3; peer review: 2 approved]. *F1000Research* (2019) 6: doi: 10.12688/f1000research.11622.3

6. Melville J. uwot: The Uniform Manifold Approximation and Projection (UMAP) Method for Dimensionality Reduction. (2019)0.2.2. doi: 10.32614/CRAN.package.uwot

7. Wickham H, Chang W, Henry L, Pedersen TL, Takahashi K, Wilke C, Woo K, Yutani H, Dunnington D, Van Den Brand T. ggplot2: Create Elegant Data Visualisations Using the Grammar of Graphics. (2007)3.5.1. doi: 10.32614/CRAN.package.ggplot2

**Antigen**	**Clone**	**Fluorophore**	**Source**	**Dilution**	**Staining**	**Panel**
TCF-1	7F11A10	PE	BioLegend	1/100	Intracellular	C
TCRγδ	B1	APC	BioLegend	1/50	Surface	F (Lineage)
TCRγδ	B1	BV421	BioLegend	1/50	Surface	A,B,C,D, E (Lineage)
TCRγδ	B1.1	FITC	eBioscience	1/30	Surface	Cytokines
